# Low mortality rates among critically ill adults with COVID‐19 at three non‐academic intensive care units in south Sweden

**DOI:** 10.1111/aas.13972

**Published:** 2021-09-05

**Authors:** Knut Taxbro, Andreas Granath, Ola Sunnergren, Stefanie Seifert, Milena N. Jakubczyk, Magnus Persson, Anneli Hammarskjöld, Catarina Alkemark, Fredrik Hammarskjöld, the RJL COVID‐19 Research Group

**Affiliations:** ^1^ Department of Anaesthesia and Intensive Care Medicine Ryhov County Hospital Jonkoping Sweden; ^2^ Department of Biomedical and Clinical Sciences Linköping University Linköping Sweden; ^3^ Department of Otorhinolaryngology Region Jönköping County Jonkoping Sweden; ^4^ Department of Anaesthesia and Intensive Care Medicine Eksjö Hospital Eksjö Sweden; ^5^ Department of Anaesthesia and Intensive Care Medicine Värnamo Hospital Värnamo Sweden

**Keywords:** acute respiratory distress syndrome, COVID‐19, intensive care, ventilation

## Abstract

**Background:**

The COVID‐19 pandemic has put an exceptional strain on intensive care units worldwide. During the first year, the survival of patients with acute hypoxaemic respiratory failure appears to have improved. We aimed to describe the mortality rates, management characteristics and two pandemic waves during the first year at three non‐academic rural intensive care units in Sweden.

**Methods:**

We retrospectively analysed all cases of COVID‐19 admitted to intensive care units in Region Jönköping County during 1 year. The primary endpoint was 30‐day mortality.

**Results:**

Between 14th March 2020 and 13th March 2021, 264 patients were admitted to undergo intensive care with confirmed SARS‐CoV‐2 infection. The 30‐day mortality rate after the initial intensive care admission was 12.9%, and this rate remained unchanged during both pandemic waves. However, we found several distinct differences between the two pandemic waves, including an increase in the use of high‐flow nasal oxygen but a decrease in invasive mechanical ventilation use, biochemical markers of inflammation, continuous renal replacement therapy and length of stay in the intensive care unit.

**Conclusion:**

Our study showed that critically ill patients with COVID‐19 in Sweden have a low 30‐day mortality rate which compares well with results published from academic centres and national cohorts throughout Scandinavia. During the second pandemic wave, the proportion of patients receiving invasive mechanical ventilation and continuous renal replacement therapy was lower than that in the first wave. This could be the result of increased knowledge and improved therapeutic options.

**Editorial Comment**

This study presents 1‐year experience of managing COVID‐19 in three non‐university hospital ICUs. The authors report mortality rates and a shift in management and improved outcomes of patients as the pandemic evolved similar to those described at larger tertiary care and academic centres.

## 1 INTRODUCTION

Currently, there is an unprecedented surge in the demand for intensive care resources worldwide due to the coronavirus disease (COVID‐19) pandemic. Initial reports from China and Italy described high mortality rates among intubated, mechanically ventilated patients.^1^, ^2^ In comparison, more recent reports from Scandinavia and central Europe have shown lower 30‐day mortality rates.^3^, ^4^, ^5^, ^6^, ^7^, ^8^, ^9^ The first case of COVID‐19 in Sweden was reported in Jönköping on the 31st of January 2020 in a patient who had travelled abroad. Six weeks later, on 14 March 2020, the first patient with COVID‐19 was admitted to an intensive care department in Jönköping County.

During both waves, Jönköping County, a region with 360,000 inhabitants and three hospitals which under normal circumstances had 13 intensive care unit (ICU) beds and ventilators, had the highest number of both confirmed severe acute respiratory syndrome coronavirus 2 (SARS‐CoV‐2) positive cases and critically ill COVID‐19 patients in Sweden, resulting in an exceptionally challenging situation throughout the first year.^10^

Data from the Swedish Intensive Care Registry have indicated that risk‐adjusted mortality rates among ICU patients with COVID‐19 in Region Jönköping County have been significantly lower than those in the rest of Sweden.^11^ By contrast, previous reports have shown a beneficial effect on mortality among mechanically ventilated patients with non‐COVID‐19 acute respiratory distress syndrome (ARDS) in high‐volume academic centres compared with non‐academic units.^12^, ^13^ Little is known about the clinical characteristics and outcomes among critically ill COVID‐19 patients admitted to rural, non‐academic ICUs in Sweden. Typically, these units have a lower staff‐to‐patient ratio when compared with academic centres.^14^ Thus, it is of key interest to describe management and outcome features in non‐academic hospitals in this group of patients, particularly during a pandemic when ICU resources are scarce and many patients had to be treated in up scaled non‐academic ICUs. Another interesting aspect of the COVID‐19 pandemic was the distinct periods of high incidence (the first and second waves) with an intermediate period of two months when the ICUs in Region Jönköping County had not admitted any COVID‐19 patients. These circumstances made it possible for the ICU staff to learn from the first wave both medically and in terms of how to organise an ICU for the management of COVID‐19 patients.

We aimed to describe the mortality rates, management characteristics and two pandemic waves (W1 and W2) during the first year at three non‐academic rural intensive care units in Jönköping County, Sweden.

## 2 METHODS

### 2.1 Study design, patients and main outcomes

The present study was a retrospective analysis of all adult (age ≥18 years) patients admitted to three different ICUs at three hospitals in Region Jönköping County (Sweden) with laboratory‐verified polymerase chain reaction assay results during the first year of the COVID‐19 pandemic. We excluded postoperative patients with uncomplicated (in spontaneous ventilation) COVID‐19. Patients’ demographics included age, sex, body mass index, presence of comorbidities (cardiac disease, hypertension, diabetes, kidney/liver/lung disease, immunosuppression, neuromuscular disease and active or previous cancer), as well as present or previous smoking habits. The primary outcome was 30‐day mortality. Secondary outcomes included the length of stay (LOS) in the ICU, days on invasive mechanical ventilation (IMV), ICU and in‐hospital mortality, discharge destination and ICU‐related complications.

### 2.2 Ethical considerations

The study protocol was approved by the Swedish Ethical Review Authority on 26 August 2020 (Dnr 2020–02758). The need for informed consent was waived due to the retrospective nature of the study.

### 2.3 Definitions

The date intervals defining comparators W1 and W2 were from the 14th of March 2020 to the 13th of September 2020 and 14th of September 2020 to the 13th of March 2021, respectively.

ARDS was defined using the criteria put forth by the Swedish Intensive Care Registry adopted from the Berlin definition.^15^

Comorbidities were defined using the International Classification of Diseases and Related Health Problems, tenth revision (ICD‐10) codes in the medical records prior to index admission: cardiovascular disease (I25 and/or I50), hypertension (I10), diabetes (E10/E11), asthma (J45) and chronic obstructive pulmonary disease (J44) in combination with active treatment. Chronic liver disease was defined using the ICD‐10 codes K70–K77. Pulmonary disease was defined as emphysema (J43) and/or pulmonary fibrosis (J84). Neuromuscular disease was defined as post‐polio syndrome or multiple sclerosis (ICD‐10 codes B91, G14 or G35). All solid or haematologic malignancies (active or past) were included in the study. Immunosuppression was classified according to the simplified acute physiology 3 scoring system,^16^ and kidney function was categorised according to the Kidney Disease: Improving Global Outcomes classification of chronic kidney disease based on the glomerular filtration rate.^17^ Ventilator time was defined as positive pressure ventilation through either an endotracheal tube or tracheostomy cannula. Transfer to another ICU due to overcrowding was regarded as a continuation of the index admission. Only index admissions were included in the analysis. Any systemic use of steroids during intensive care was categorised as steroid use, irrespective of the dose, timing and duration.

### 2.4 Population size, ICU capacity and peak ICU admission rates

The total catchment population for the three hospitals (Ryhov County Hospital, Eksjö Hospital, and Värnamo hospital) was 361,759.^18^ The ICUs were closed and mixed, and led by consultant board‐certified specialists in anaesthesiology and intensive care medicine. Prior to the pandemic, the three ICUs had seven, four and four ventilator beds, respectively (4.15 ICU beds per 100,000 inhabitants) (Table 1). There was no dedicated retrieval service in this region. The three ICUs had a mutual guideline for treating COVID‐19 patients in the ICU. This document served as a platform and was updated continuously.

**TABLE 1:** Intensive care unit capacity, admission rates and staffing prior to the pandemic surge

	Ventilator beds available 24/7	Annual admission rate 2015–2019	Median SAPS 3 score 2015–2019	Proportion of patients receiving IMV 2015–2019	Specialist physicians holding full time employment	ICU nurses holding full time employment
Ryhov	7	538	59	50%	24.5	52.1
Eksjö	4	342	58	31%	6.3	21.4
Värnamo	4	515	47	12%	7.8	19.7

ICU, intensive care unit; IMV, invasive mechanical ventilation.

### 2.5 Data collection and statistical analysis

The data were collected and validated by a group of specially trained abstractors (ICU‐registered nurses) from two digital medical record systems (MetaVision^™^ and Cambio Cosmic^™^). The baseline clinical characteristics were summarised using descriptive statistics, stratified by time period (W1 and W2) of the index admission. Categorical variables are reported as frequencies and percentages and continuous variables as medians and interquartile ranges (IQRs). Differences between W1 and W2 were assessed using chi‐squared tests, Fisher's exact tests or Mann–Whitney *U* tests, as appropriate. All statistical analyses were performed using SPSS version 27 (IBM Corporation). Analysis items with *p* < .05 were considered statistically significant.

## 3 RESULTS

**FIGURE 1:**
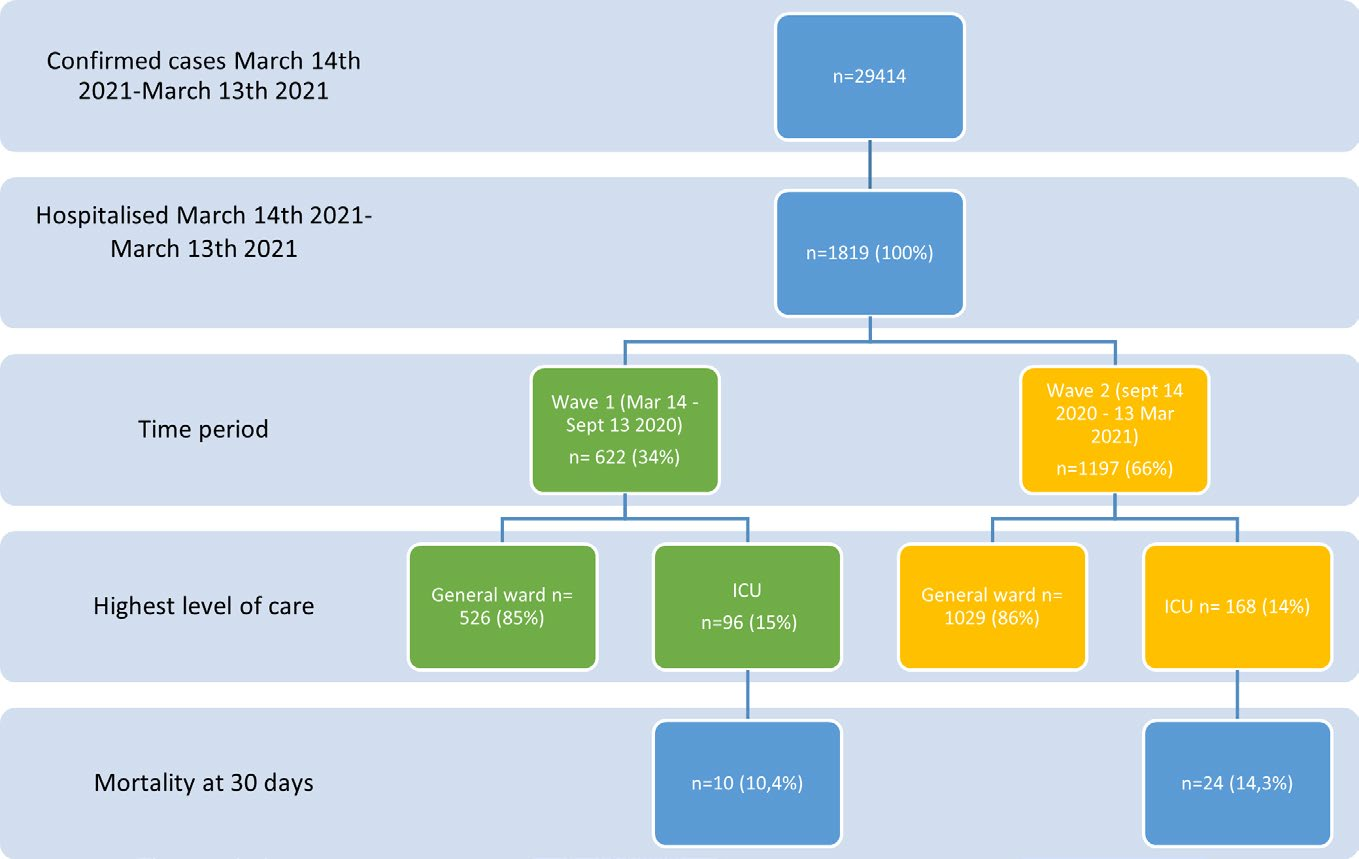
Study outline. ICU, intensive care unit [Colour figure can be viewed at wileyonlinelibrary.com]

Between 14th March 2020 and 13th March 2021, 264 patients were admitted to the ICU with confirmed SARS‐CoV‐2 infection. The baseline characteristics are shown in Table 2. A majority were male and overweight/obese, with a median age of 65 years (range, 18–87). Hypertension and diabetes were the commonest comorbidities. The median time from the onset of first symptoms to arrival at the ICU was 9 days (IQR, 7–12), and after being hospitalised for a median of 1.5 days (IQR, 0–4). An outline of patient numbers and time periods is shown in Figure 1.

**TABLE 2:** Characteristics at initial ICU admission among all patients with COVID‐19

	First wave	Second wave	*p*‐value	Total population
Patients admitted to ICU (%)	96 (36)	168 (64)		264 (100)
Age. years (range)	63 (19–86)	67 (14–87)	−7.0 to 0.5*	65 (18–87)
Sex (%)			.3	
Male	64 (66)	123 (73)	‐	187 (70)
Female	32 (34)	45 (27)	‐	77 (30)
BMI kg/m^2^ (range)	29 (20–56)	29 (17–51)	.9	29 (7)
Underweight (<18.5 kg/m^2^) (%)	− (−)	*1 (0.6)*	‐	*1 (0.4)*
Normal (18.5–24.9 kg/m^2^) (%)	*10 (10.4)*	*22 (13.1)*	‐	*32 (12.1)*
Overweight (25.0–29.9 kg/m^2^) (%)	*38 (39.6)*	*62 (36.9)*	‐	*100 (37.9)*
Obese (>30.0 kg/m^2^) (%)	*47 (49.0)*	*78 (46.4)*	‐	*125 (47.0)*
Missing data (%)	*1 (1.0)*	*5 (3.0)*	‐	*6 (2.3)*
Chronic cardiac disease (%)	19 (20.0)	35 (21.0)	.8	54 (20.5)
Hypertensive disease (%)	48 (50.0)	98 (58.3)	.2	146 (55.3)
Asthma (%)	14 (14.6)	22 (13.1)	.7	36 (13.6)
COPD (%)	7 (7.3)	16 (9.5)	.5	23 (8.7)
Smoker (%)	26 (27.0)	50 (29.7)	.3	76 (28.8)
Pulmonary disease (%)	13 (13.5)	12 (7.1)	.09	25 (9.5)
Immunosuppression (%)	11 (4.2)	18 (6.9)	1.0	29 (11.0)
Chronic liver disease (%)	1 (0.4)	4 (1.5)	.7	5 (1.9)
Chronic renal failure KDIGO grade (%)			.3	
G1	48 (50)	63 (37.5)	‐	111 (42.0)
G2	35 (36.5)	68 (40.5)	‐	103 (39.0)
G3a	9 (9.4)	22 (13.1)	‐	31 (11.7)
G3b	3 (3.1)	12 (7.1)	‐	15 (5.7)
G4	1 (1.0)	3 (1.8)	‐	4 (1.5)
Diabetes	26 (27.0)	52 (30.1)	.5	78 (29.5)
Type 1 (%)	4 (4.2)	4 (2.4)	‐	8 (3.0)
Type 2 (%)	22 (23.0)	48 (28.6)	‐	70 (26.5)
Neuromuscular disease (%)	6 (2.3)	3 (1.1)	.08	9 (3.4)
Active malignancy (%)	6 (6.3)	12 (7.2)	.4	18 (6.8)
Solid tumour (%)	5 (5.3)	7 (4.1)	‐	12 (4.5)
Haematologic cancer (%)	1 (1.0)	5 (3.0)	‐	6 (2.3)
Index admission from			.06	
Emergency department	14 (14.6)	39 (23.2)	‐	‐53 (20.3)
COVID 19 ward	67 (69.8)	114 (67.9)	‐	181(68.6)
Non‐COVID 19 ward	14 (14.6)	10 (6.0)	‐	24 (9.1)
Another ICU	1 (1.0)	2 (1.2)	‐	3 (1.1)
Missing	0 (0)	3 (1.8)	‐	3 (1.1)
Index admission to			.1	
Ryhov County Hospital	53 (55.2)	81 (48.2)	‐	134 (50.7)
Värnamo Hospital	23 (24.0)	32 (19.2)	‐	55 (20.8)
Eksjö Hospital	20 (20.8)	55 (32.7)	‐	75 (28.4)

The significance level refers to the distribution of for example ARDS classes between the two waves, and not between the specific groups (in italics).

BMI, body mass index; COPD, chronic obstructive pulmonary disease; ICU, intensive care unit; G. grade: *95% confidence interval.

The highest occupancy rates in the three ICUs occurred during W1 and were 257%, 225% and 225% of the normal capacities of the three hospitals. Notably, there was no difference in the proportion of patients admitted to the ICU directly from the emergency department (14.6% during W1vs. 23.2% during W2).

Detailed characteristics of ICU management are given in Table 3. The proportion of patients who received high flow nasal oxygen (HFNO) administered as the highest level of respiratory support in the ICU increased from 7.3% to 20.8% (*p* = .004) in W2 compared with W1. In comparison, the proportion of patients who underwent IMV was lower in W2 than in W1 (72.6% and 85.4%, *p* = .017). Similarly, the proportions of patients who were treated with prone ventilation (75.6% vs. 58.0%, *p* = .01) and prolonged infusion (>24 h) of neuromuscular blockade (75.6% vs. 42.0%, *p* < .001) were lower during W2.

**TABLE 3:** Characteristics of ICU management among all patients with COVID‐19

	First wave	Second wave	*p* value	Total population
Number of patients admitted to ICU (%)	96 (36)	168 (64)		264 (100)
Days in hospital prior to ICU admission (IQR)	2.0 (1–5)	1.0 (0–4)	.3	1.5 (0–4)
Days between first symptom and ICU admission (IQR)	10.0 (7–12)	9.0 (7–12)	.2	9 (7–12)
Limitation of care during ICU admission (%)	19 (19.8)	34 (20.2) (Missing data n=1)	.9	53 (20.1)
SAPS3 score on index ICU admission (IQR)	57 (48–62)	58 (51–64)	.5	58 (49–63)
Highest level of respiratory support				
Oxymask	6 (6.3)	11 (6.6)	.9	17 (6.4)
HFNO (%)	7 (7.3)	35 (20.8)	.004	42 (15.9)
NIV (%)	1 (1.0)	0 (0)	–	1 (0.4)
IMV (%)	82 (85.4)	122 (72.6)	.017	204 (77.3)
ARDS class* (among patients who underwent IMV)	82 (40.8)	119 (59.2)	.8	201 (100)^a^
Mild. (P/F ratio >26.6 kPa) (%)	*8 (9.8)*	*15 (12.6)*	‐	*23 (11.4)*
Moderate. P/F ratio >13.3 and ≤26.6 kPa (%)	*59 (72.0)*	*82 (68.9)*	‐	*141 (70.1)*
Severe. P/F ratio ≤13.3 kPa) (%)	*15 (18.3)*	*22 (18.5)*	‐	*37 (18.4)*
Prone ventilation (%) (among patients with ARDS who underwent IMV)	62 (75.6)	69 (58.0)	.01	131 (65.2)
Hours in prone ventilation (IQR)	31 (4–68)	13 (0–35)	.034	16 (52)
NMB >24 h (%) (among patients with ARDS who underwent IMV)	62 (75.6)	50 (42.0)	<.001	112 (55.7)
Tracheotomy (%) (among patients with ARDS who underwent IMV)	55 (67.1)	65 (54.6)	.052	120 (58.8)
Days to tracheotomy (IQR)	9 (6–10)	7 (5–9)	.002	8 (6–10)
ECMO (%)	0 (0)	1 (0.6)	‐	1 (0.4)
CRRT (%)	26 (27.0)	16 (9.5)	<.001	42 (15.9)
Hours in CRRT (IQR)	117 (75–261)	187 (84–586)	.3	142 (75–415)
CVC (%)	89 (92.7)	153 (91.1)	.6	242 (91.7)
Vasopressor^c^ (%)			<.001	
No vasopressor	14 (14.6)	54 (32.5)	‐	68 (26.0)
<0.1 µg/kg/min	55 (57.3)	87 (52.4)	‐	142 (54.2)
>0.1 µg/kg/min	27 (28.1)	25 (15.1)	‐	52 (19.8)
TVPM (%)	1 (1.0)	4 (2.4)	.7	5 (1.9)
Transfer to another ICU	20 (20.8)	40 (23.8)	.6	60 (22.7)
Pharmacological treatment				
Steroids (%)	54 (56.3)	162 (96.4)	<.001	216 (81.8)
Remdesivir (%)	1 (1.0)	19 (11.3)	.002	20 (7.6)
Antibiotics (%)	85 (88.5)	146 (86.9)	.7	231 (87.5)
Tocilizumab (%)	3 (3.1)	3 (1.8)	.5	6 (2.3)
Anakinra (%)	0 (0)	0 (0)	–	0 (0)
Hydroxychloroquine/chloroquine phosphate (%)	8 (8.3)	0 (0)	<.001	8 (3.0)
Inhaled vasodilators	19 (19.8)	23 (13.7)	.2	42 (15.9)
LMWH* (%)	94 (97.9)	159 (94.6)	.2	253 (95.8)
LMWH*>9000 U/day (%)	42 (43.8)	133 (79.2)	<.001	175 (66.5)
Laboratory tests (peak value during ICU admission)				
NTproBNP ng/L (IQR)	1091 (342–3919)	589 (298–1609)	0.1	672 (307–2280)
Creatinine µmol/L (IQR)	92 (75–232)	78 (61–107)	.039	82 (52–123)
Myoglobin µg/L (IQR)	467 (183–1647)	145 (75–338)	<.001	210 (26–545)
C‐reactive protein mg/L (IQR)	271 (260–334)	148 (80–234)	<.001	197 (98–279)
Ferritin µg/L (IQR)	1365 (720–2885)	1101 (332–2922)	.8	1129 (450–2904)
Procalcitonin µg/L (IQR)	1.6 (0.6–5.4)	0.4 (0.2–1.4)	<.001	0.5 (0.2–2)

The significance level refers to the distribution of for example ARDS classes between the two waves, and not between the specific groups (in italics).

ARDS, acute respiratory distress syndrome; CRRT, continuous renal replacement therapy; CVC, central venous catheter; ECMO, extracorporeal membrane oxygenation; HFNO, high flow nasal oxygen; ICU, intensive care unit; IMV, invasive mechanical ventilation; IQR, interquartile range; LMWH, low molecular weight heparin (*tinzaparin); NIV, non‐invasive ventilation; SAPS3, simplified acute physiological score 3; TVPM, transvenous pacemaker.

^a^ Three mechanically ventilated patients admitted to the ICU for postoperative reasons were excluded from the analysis.

^b^ The lowest consistent P/F‐ ratios were recorded and used for the ARDS classification.

^c^ Noradrenaline (missing data for two patients).

Eleven patients (4.1%) were managed without HFNO, non‐invasive ventilation or IMV. Of these, eight were admitted to the ICU for reasons other than acute hypoxaemic respiratory failure (i.e., shock, liver failure, seizures, diabetic ketoacidosis, left ventricular failure, or electrolyte disturbances).

Whereas the median time for patients receiving continuous renal replacement therapy (CRRT) remained unchanged (117 vs. 187 h, *p* = .3), the proportion of patients requiring CRRT declined from 27.0% to 9.5% (*p* < .001) during W2.

The median LOS in the ICU was 10 days (IQR, 3–19), but significantly shorter (13.5 vs. 7.5 days, *p* < .001) during W2. Similarly, the median time spent in IMV decreased from 15 days to 9 days (*p* = .03).

Sixty patients (22.7%) were transferred to another ICU during the first year due to overcrowding, and the proportions were similar between waves (20.8% vs. 23.8%, *p* = .6). There was no difference in 30‐day mortality between patients who underwent inter‐facility transfers and those who did not (*p* = .3).

The rate of steroid use during intensive care increased from 54.6% during W1 to 96.4% during W2 (*p* < .001), whereas the rate of antibiotic administration remained unchanged throughout the study period (88%). Peak laboratory values of creatinine, myoglobin, C‐reactive protein, and procalcitonin were significantly lower during W2.

More than one in three patients (31.4%) experienced pressure sores; however, the rate was significantly lower during W2, from 47.9% during W1 to 22.0% (*p* < .001). The 30‐day mortality rate (Table 4) remained unchanged over the study period, with a total of 34 deaths (12.9%) recorded. Of these, 26 (9.8%) died in the ICU. In total, 43 patients (16.3%) died during hospitalisation. There were no differences in 30‐day mortality rate between W1 and W2 at any of the centres. Seven (2.7%) patients were still hospitalised at the time of the data cut‐off. Among patients with IMV, 25 (12.4%) died within 30‐days of ICU admission.

**TABLE 4:** Patient outcomes

	First wave	Second wave	*p*‐value	All
Death within 30 days (%) after index admission	10 (10.4)	24 (14.3)	.4	34 (12.9)
Death in the ICU (%)	10 (10.4)	16 (9.5)	.8	26 (9.8)
Death during hospitalisation (%)	13 (13.5)	30 (17.9)	.4	43 (16.3)
LOS (days) in ICU (IQR)	13.5 (6–22)	7.5 (2–8)	<.001	10 (3–19)
Time in IMV (IQR)	15 (9–22)	9 (5–19)	.03	12 (6–20)
ICU readmission (%)	9 (9.4)	11 (6.5)	.4	20 (7.6)
Length of hospitalisation (IQR)	32 (16–47)	20.5 (13–36)	.014	23 (14–41)
Pressure sore (%)	46 (47.9)	37 (22.0)	<.001	83 (31.4)
Heart failure (%)	19 (19.8)	26 (15.5)	.4	45 (17.3)
Cardiac arrest with CPR (%)	5 (5.2)	4 (2.4)	.2	9 (3.4)
Pneumothorax/subcutaneous emphysema/pneumomediastinum (%)	6 (6.3)	4 (2.4)	.1	10 (3.8)
Septic shock (%)	0 (0)	9 (5.4)	.03	9 (3.4)
DVT (%)	2 (2.5)	2 (0.8)	.6	4 (1.5)
PE (%)	4 (5.0)	10 (5.9)	1.0	14 (5.3)
Reintubation	8 (10)	14 (11.5)	.7	22 (10.9)
Recannulation	2 (3.0)	7 (10.8)	.3	9 (7.5)
RRT 30 days after initial ICU admission (%)	7 (7.3)	1 (0.6)	.7	8 (3.0)
Discharge destination				
Home (%)	70 (72.9)	110 (65.5)		180 (68.2)
Nursing home (%)	12 (12.5)	19 (11.3)		31 (11.7)
Hospitalised at time of data cut‐off (%)	0 (0)	7 (4.2)	–	7 (2.7)
Deceased at time of data cut‐off (%)	14 (14.6)	32 (19.0)	–	46 (17.4)

ICU, intensive care unit; LOS, length of stay; IQR, interquartile range; IMV, invasive mechanical ventilation; CPR, cardiopulmonary resuscitation; DVT, deep vein thrombosis; PE, pulmonary embolus; RRT, renal replacement therapy.

## 4 DISCUSSION

To the best of our knowledge, the present study represents the first complete analysis of a 1‐year COVID‐19 ICU cohort in Scandinavia. The main findings were the low 30‐day and in‐hospital mortality rates that compared well with the results from both Scandinavian tertiary centre ICUs and national data sets during W1.^3^, ^4^, ^5^, ^6^, ^7^, ^8^ Furthermore, the mortality rates were consistently low throughout both pandemic waves during the first year.

IMV in combination with prone positioning is a cornerstone in the treatment of moderate‐to‐severe ARDS, and these measures were frequently used during both waves.^19^ The overall proportion of patients with IMV (77%) was slightly lower than that reported at other Scandinavian centres according to data from the initial pandemic phase. By contrast, the rate of use of prone positioning during IMV was substantially higher in this study.^3^, ^4^, ^5^, ^6^, ^7^, ^8^, ^19^ In a Swedish report published by Chew et al., 40% of patients had severe ARDS, which is roughly twice that reported by Laake et al. in Norway and the present study. ARDS severity correlates well with mortality and could explain some of the differences between the studies.^20^, ^21^

The LOS in the ICU is a relevant factor during times of exceptional strain on limited ICU resources. With the exception of the results of a smaller, single‐centre cohort study conducted in Norway,^8^ the LOS in the ICU compared well within Scandinavia.^3^, ^4^, ^5^, ^6^, ^7^ By contrast, in the present study, the median LOS was slightly shorter, with a median of 10 days (IQR, 3–19). First, a feasible explanation could be our liberal approach to tracheostomies (60%) in combination with the possibility of discharging tracheotomised patients to intermediate care (non‐ICU) wards. These findings illustrate the potential clinical benefits (easier weaning and early discharge) of early tracheostomy, and agree with those of studies other than those recommended by international guidelines available in the initial phase of the pandemic.^22^, ^23^, ^24^ Second, we believe that sedation levels might have been lower during W2 as a result of increased intensive care experience among the nurse anaesthetists allocated to the COVID‐19 ICUs.

The venous thromboembolism (VTE) rate has been reported to be as high as 31% (95% confidence interval [CI], 23–39) among ICU patients with COVID‐19, and the corresponding number of clinically significant cases of VTE in the present study was 6% (95% CI, 4–10).^25^ The observed VTE rate could reflect the widespread (66%) prophylactic use of low molecular weight heparin at doses higher than those typically used for prophylaxis. However, the evidence supporting different dose levels of anticoagulation in COVID‐19 is controversial,^26^, ^27^, ^28^ and in an attempt to minimise patient harm, we closely monitored the levels of anti‐factor Xa.

The overall use of steroids was relatively high during W1 (54.6%) and very high (>95%) during W2. We believe that the increased use of steroids reflects the rapid adaptation to novel findings regarding the efficacy of steroids as a treatment for severe COVID‐19.^29^ By contrast, the use of more experimental treatments (tocilizumab, anakinra and chloroquine) which remained limited throughout the year, reflecting conflicting evidence and our conservative approach.^30^

Collectively, we followed a simple treatment protocol throughout the year. The goal of this protocol (Supplementary Files) was to reflect the current knowledge, and the protocol was updated on several occasions. In addition to pharmacotherapy, the protocol included suggested ventilator strategies, positioning guidance, sedation strategies, staffing requirements, guidance for microbiological sampling (bronchoalveolar lavage) and hygiene routines. This protocol could have helped harmonise the approaches of the staff to the management of this novel patient group according to the best available evidence, and in maintaining the ‘less‐is‐more’ strategy, focussing on getting the basics right, every time.

Interestingly, during W2, we noted a decline in the use of IMV, prone positioning and the prolonged use of neuromuscular blockers without changes in the mortality rate. Among patients who underwent IMV, the distribution of ARDS classes remained unchanged; however, more patients with low P/F ratios were managed without IMV during W2. These findings, together with the decreased LOS and incidence of pressure ulcers, are, in our opinion, a result of several factors. These include higher levels of knowledge about the disease, successively more trained staff, team‐training practices, and no shortage of medications and disposables. Furthermore, steroid and anticoagulation therapy prior to ICU admission may have contributed to the difference in the need for IMV. This could be supported by the biochemical findings with lower median peak values of both C‐reactive protein and procalcitonin.

A substantial number of patients were transferred to another ICU, mainly due to the limited capacity of the referring unit. Previously, some data have indicated that patients exposed to transfer have an increased risk of death.^31^ On the contrary, some reports have indicated a lower mortality rate among COVID‐19 patients transferred between ICUs.^6^, ^32^, ^33^

The present study had several strengths. First, no patient was lost to follow‐up with regard to the primary endpoint. Second, we present a complete, high‐resolution dataset from the first pandemic year from ICUs in three non‐academic hospitals. Third, the data were collected and validated by trained abstractors using the same electronic patient data management and chart systems.

The main limitation of the present study lies in its retrospective nature. Although a prospective study could increase the validity of our findings, given the rapid evolution of the pandemic, there was simply no time to plan for such a study beforehand. Further, we did not have data on patient ethnicity, socioeconomic status and ICU staffing throughout the year. These factors are relevant and known confounders of mortality among critically ill patients and must be considered when comparing variations in mortality between units.^34^, ^35^, ^36^, ^37^ Finally, a substantial proportion of the patients were transferred outside the region and details regarding laboratory values, time spent in CRRT and prone positioning, and complications could be incomplete.

In conclusion, the present study showed that critically ill patients in our county with COVID‐19 have a low 30‐day mortality rate, which compared well with results from academic centres,^3^, ^5^ and national cohorts in Scandinavia.^4^, ^7^, ^8^

## CONFLICTS OF INTEREST

The authors declare that they have no conflicts of interest.

## Supporting information

Supplementary Material

Supplementary Material

## Data Availability

The data that support the findings of this study are available from the corresponding author upon reasonable request.

## References

[1] Grasselli G , Zangrillo A , Zanella A , et al. Baseline characteristics and outcomes of 1591 patients infected with SARS‐CoV‐2 admitted to ICUs of the Lombardy region, Italy. JAMA. 2020;323(16):1574‐1581. 10.1001/jama.2020.5394. 32250385 PMC7136855

[2] Yang X , Yu Y , Xu J , et al. Clinical course and outcomes of critically ill patients with SARS‐CoV‐2 pneumonia in Wuhan, China: a single‐centered, retrospective, observational study. Lancet Respir Med. 2020;8:475‐481. 32105632 10.1016/S2213-2600(20)30079-5 PMC7102538

[3] Søvik S , Bådstøløkken PM , Sørensen V , et al. A single‐centre, prospective cohort study of COVID‐19 patients admitted to ICU for mechanical ventilatory support. Acta Anaesthesiol Scand. 2021;65:351‐359. 33128800 10.1111/aas.13726 PMC7894152

[4] Ahlström B , Frithiof R , Hultström M , Larsson IM , Strandberg G , Lipcsey M . The swedish covid‐19 intensive care cohort: risk factors of ICU admission and ICU mortality. Acta Anaesthesiol Scand. 2021;65:525‐533. 10.1111/aas.13781 PMC8013403 33438198

[5] Larsson E , Brattström O , Agvald‐Öhman C , et al. Karolinska intensive care COVID‐19 Study Group. Characteristics and outcomes of patients with COVID‐19 admitted to ICU in a tertiary hospital in Stockholm, Sweden. Acta Anaesthesiol Scand. 2021;65:76‐81. 32892337 10.1111/aas.13694 PMC7756749

[6] Chew MS , Blixt PJ , Åhman R , et al. National outcomes and characteristics of patients admitted to Swedish intensive care units for COVID‐19: a registry‐based cohort study. Eur J Anaesthesiol. 2021;38:335‐343. 33534266 10.1097/EJA.0000000000001459

[7] Laake JH , Buanes EA , Småstuen MC , et al. A prospective observational study. Acta Anaesthesiol Scand. 2020;2021(65):618‐628. 10.1111/aas.13785 PMC8014826 33501998

[8] Haase N , Plovsing R , Christensen S , et al. Characteristics, interventions, and longer term outcomes of COVID‐19 ICU patients in Denmark—a nationwide, observational study. Acta Anaesthesiol Scand. 2021;65:68‐75. 32929715 10.1111/aas.13701

[9] COVID‐ICU Group on behalf of the REVA Network and the COVID‐ICU Investigators . Clinical characteristics and day‐90 outcomes of 4244 critically ill adults with COVID‐19: a prospective cohort study. Intensive Care Med 2021;47:60‐73. 33211135 10.1007/s00134-020-06294-x PMC7674575

[10] The Swedish Public Health Agency . COVID‐19 cases in Sweden; 2021. Available online: https://experience.arcgis.com/experience/09f821667ce64bf7be6f9f87457ed9aa, accessed on 27/05/2021.

[11] Swedish Intensive Care Registry . Data portal for The Swedish Intensive Care Registry, 2021. Available online: https://portal.icuregswe.org/utdata/sv/home, accessed on 27/05/2021.

[12] Kahn JM , Goss CH , Heagerty PJ , Kramer AA , O'Brien CR , Rubenfeld GD . Hospital volume and the outcomes of mechanical ventilation. N Engl J Med. 2006;355:41‐50. 16822995 10.1056/NEJMsa053993

[13] Ike JD , Kempker JA , Kramer MR , Martin GS . The association between acute respiratory distress syndrome hospital case volume and mortality in a U.S. Cohort, 2002–2011. Crit Care Med. 2018;46(5):764‐773. 29394181 10.1097/CCM.0000000000003015 PMC5914522

[14] Swedish Association for Anaesthesia and Intensive Care and Swedish Intensive Care Association (2015). Guidelines for Swedish intensive care. Available online: https://sfai.se/wp‐content/uploads/2015/02/Riktlinjer‐Svensk‐Intensivv%C3%A5rd_‐rev‐2015.pdf [accessed on 27/05/2021].

[15] Swedish Intensive Care Registry . SIR Diagnostic criteria; 2021. Available online: https://portal.icuregswe.org/utdata/sv/home, accessed on 27/05/2021.

[16] The SAPS 3 Research Group (2021). Publications. Available online: www.saps3.org [accessed on 27/05/2021].

[17] KDIGO Clinical Practice Guideline for Acute Kidney Injury (2016). Guidelines. Available online: https://kdigo.org/wp‐content/uploads/2016/10/KDIGO‐2012‐AKI‐Guideline‐English.pdf [accessed on 27/05/2021].

[18] Statistics Sweden (2019). Population data. Available online: https://www.scb.se/hitta‐statistik/statistik‐efter‐amne/befolkning/befolkningens‐sammansattning/befolkningsstatistik/pong/tabell‐och‐diagram/kvartals‐‐och‐halvarsstatistik‐‐kommun‐lan‐och‐riket/kvartal‐1‐2019/ [accessed on 07/05/2021].

[19] Thompson BT , Chambers RC , Liu KD . Acute respiratory distress syndrome. N Engl J Med. 2017;377:562‐572. 28792873 10.1056/NEJMra1608077

[20] Bellani G , Laffey JG , Pham T , et al. Epidemiology, patterns of care, and mortality for patients with acute respiratory distress syndrome in intensive care units in 50 countries. JAMA. 2016;315:788‐800. 26903337 10.1001/jama.2016.0291

[21] Lewandowski K , Metz J , Deutschmann C , et al. Incidence, severity, and mortality of acute respiratory failure in Berlin, Germany. Am J Respir Crit Care Med. 1995;151:1121‐1125. 7697241 10.1164/ajrccm.151.4.7697241

[22] Kwak PE , Connors JR , Benedict PA , et al. Early outcomes from early tracheostomy for patients with COVID‐19. JAMA Otolaryngol Head Neck Surg. 2021;147:239‐244. 33331855 10.1001/jamaoto.2020.4837 PMC7747038

[23] Sommer DD , Engels PT , Weitzel EK , et al. Recommendations from the CSO‐HNS taskforce on performance of tracheotomy during the COVID‐19 pandemic. J Otolaryngol Head Neck Surg. 2020;49:23. 32340627 10.1186/s40463-020-00414-9 PMC7184547

[24] Givi B , Schiff BA , Chinn SB , et al. Safety recommendations for evaluation and surgery of the head and neck during the COVID‐19 pandemic. JAMA Otolaryngol Head Neck Surg. 2020;146:579‐584. 32232423 10.1001/jamaoto.2020.0780

[25] Malas MB , Naazie IN , Elsayed N , Mathlouthi A , Marmor R , Clary B . Thromboembolism risk of COVID‐19 is high and associated with a higher risk of mortality: a systematic review and meta‐analysis. EClinicalMedicine. 2020;29:100639. 33251499 10.1016/j.eclinm.2020.100639 PMC7679115

[26] Al‐Samkari H , Gupta S , Leaf RK , et al. Thrombosis, bleeding, and the observational effect of early therapeutic anticoagulation on survival in critically Ill patients with COVID‐19. Ann Intern Med. 2021;174:622‐632. 33493012 10.7326/M20-6739 PMC7863679

[27] Rentsch CT , Beckman JA , Tomlinson L , et al. Early initiation of prophylactic anticoagulation for prevention of coronavirus disease 2019 mortality in patients admitted to hospital in the United States: cohort study. BMJ. 2021;372:n311. 33574135 10.1136/bmj.n311 PMC7876672

[28] The REMAP‐CAP, ACTIV‐4a, and ATTACC Investigators (2021). Therapeutic Anticoagulation in Critically Ill Patients with Covid‐19 – Preliminary Report. Available online: https://www.medrxiv.org/content/10.1101/2021.03.10.21252749v1.full.pdf [accessed on 25/05/2021].

[29] WHO Rapid Evidence Appraisal for COVID‐19 Therapies (REACT) Working Group . Sterne JAC , Murthy S , et al. Association between administration of systemic corticosteroids and mortality among critically ill patients with COVID‐19: a meta‐analysis. JAMA 2020;324:1330‐1341. 32876694 10.1001/jama.2020.17023 PMC7489434

[30] Attaway AH , Scheraga RG , Bhimraj A , Biehl M , Hatipoğlu U . Severe COVID‐19 pneumonia: pathogenesis and clinical management. BMJ. 2021;372:n436. 33692022 10.1136/bmj.n436

[31] ESICM LIVES 2019: Berlin, Germany. 28 September ‐ 2 October 2019. Intensive Care Med Exp 2019;7:55. 10.1186/s40635-019-0265-y PMC6763544 31559498

[32] Guillon A , Laurent E , Godillon L , Kimmoun A , Grammatico‐Guillon L . Inter‐regional transfers for pandemic surges were associated with reduced mortality rates. Intensive Care Med. 2021;47(7):798‐800. 10.1007/s00134-021-06412-3. 33991207 PMC8122204

[33] Painvin B , Messet H , Rodriguez M , et al. Inter‐hospital transport of critically ill patients to manage the intensive care unit surge during the COVID‐19 pandemic in France. Ann Intensive Care. 2021;11:54. 33788010 10.1186/s13613-021-00841-5 PMC8011063

[34] West E , Barron DN , Harrison D , Rafferty AM , Rowan K , Sanderson C . Nurse staffing, medical staffing and mortality in intensive care: An observational study. Int J Nurs Stud. 2014;51:781‐794. 24636667 10.1016/j.ijnurstu.2014.02.007

[35] McGahan M , Kucharski G , Coyer F . Best WACCCN Nursing Review Paper 2011 sponsored by Elsevier. Nurse staffing levels and the incidence of mortality and morbidity in the adult intensive care unit: a literature review. Aust Crit Care. 2012;25:64‐77. 22515951 10.1016/j.aucc.2012.03.003

[36] Berkowitz SA , Cené CW , Chatterjee A . Covid‐19 and health equity ‐ time to think big. N Engl J Med. 2020;383:e76. 32706955 10.1056/NEJMp2021209

[37] Mathur R , Rentsch CT , Morton CE , et al. OpenSAFELY Collaborative. Ethnic differences in SARS‐CoV‐2 infection and COVID‐19‐related hospitalisation, intensive care unit admission, and death in 17 million adults in England: an observational cohort study using the OpenSAFELY platform. Lancet. 2021;397:1711‐1724. 33939953 10.1016/S0140-6736(21)00634-6 PMC8087292

