# Supplementary material for: Low mortality rates among critically ill adults with COVID‐19 at three non‐academic intensive care units in south Sweden
Source: Acta Anaesthesiol Scand. 2021 Sep 5;65(10):1457–65. doi: 10.1111/aas.13972 (PMC8441887; doi:10.1111/aas.13972)
Supplement: Supplementary file 1 — Supplementary Material [file AAS-65-1457-s001.docx]

#
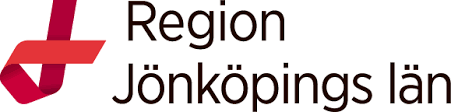


# **Treatment strategies for patients with**

# **Covid-19**

# **Department of Anaesthesia and Intensive Care Medicine,**

# **Ryhov County Hospital,**

a bedside suggestion.

Version 3.4: 2021-05-03

Author: Fredrik Hammarskjöld, Senior Consultant, Associate Professor

Approved by: Thomas Öhman, Senior Consultant, Head of the ICU

Department of Anaesthesia and Intensive Care Medicine

Ryhov County Hospital

Jönköping, Sweden

**Background:**

Patients with severe Covid-19 infections in the ICU involves new challenges. Unfortunately, there is very limited high-level evidence regarding treatment-recommendations. These guidelines are, from the beginning, based on experiences from other countries. Gradually, we have been able to include our own experience and knowledge, from Sweden and Jönköping.

The lack of high-degree evidence does not justify the use of potentially positive drugs and treatment strategies - *Primum non nocere.* Our philosophy is to deliver safe and well-established ICU treatment strategies as long as possible.

This document nwill be very dynamic due to the enormous inflow of published studies (most often case- studies or limited cohort studies, predominately retrospective) ~~this document will be very dynamic~~ and an updated version will always be available on our departments homepage. When a new version is presented, it will be sent e-mailed to all staff at our unit. Text in red reveals changes from the previous version.

There are, most often, several ways to solve a problem. The initial results for treating COVID-19 patients, in our ICU have until now been very promising. We believe that one of the most important factors is high adherence to these recommendations and the ability to solve new problems. Furthermore, to increase patient-safety and reduce the stress for the staff- simplicity and conformity are key factors. This cannot be stressed enough since a high degree of our covid-staff does not have specific ICU education and experience. Very advanced and technically complicated treatments should be avoided, but could be used when necessary.

If you have specific viewpoints or comments on these guideline, please send an e-mail to the author.

**Who should be treated in the ICU?**

When considering a patient with Covid-19 for critical care standard assessments should be made. All Covid-19 positive patients treated in the ward outside the ICU are on daily basis discussed with the responsible physicians for potential ICU treatment.

It is of great importance that a patient where intensive care is indicated is transferred to the ICU early in the case of respiratory deterioration, i.e. SaO_2_ < 90% (mask O_2_ ≥8 l/min) or pCO_2_ >6 kPa or an increase in respiratory rate reaching around 30 breaths per min. Assessing Covid-19 patients’ respiratory distress is difficult almost treacherous, as they often feel comfortable despite severe respiratory hypoxic failure- *happy hypoxia.*

Other factors that increase the indication for ICU-treatment are: confusion, decreased level of consciousness, decreased urine production despite fluid treatment, arrhythmias (i.e. atrial fibrillation), circulatory instability or chronic diseases that lower the threshold for critical care admission.

Do not let the patients get exhausted on the wards or the emergency department. Exhaustion will increase the risk for complications during the intubation procedure. We have noticed several patients with pneumothorax caused by very strenuous respiratory effort. There is growing evidence regarding risk factors for severe Covid-19 disease and mortality. These are probably increasing age, frailty, low physical capacity, cardio-vascular diseases, hypertension, high BMI, type 2 diabetes, COPD, smoking, compromised immune system, organ transplantation, treatment with immune-depressants (i.e. Rituximab), and male sex.

*However, every single patient must be assessed individually and according to his or her own risk factors considering chronic diseases, biological age, physical capacity, and their own opinion about ICU-treatment. Possible treatment limitations should always be documented according to our department routines.*

**THE FOLLOWING LABORATORY-FINDINGS INDICATE THE RISK FOR SEVERE ILLNESS AND INCREASED MORTALITY**

- CRP >100 mg/l without signs of simultaneous bacterial infection
- Gradually increasing LD >8 mikrokat/l
- Increasing creatinine, >100 mikromol/l in previous healthy persons
- Increase D-dimer >1 mg/l FEU
- Troponin T >15 nanog/L
- Low lymphocytes (with normal or high leukocytes)
- S-Ferritin >1000 microg/l

**LABORATORY SAMPLES AND OTHER EXAMINATIONS:**

Chemistry:

Use the pre-specified special batches for Covid-19 in the laboratory system, both at arrival and on daily basis.

Microbiology:

*At arrival:*

- Blood cultures
- Intubated patients: Bronchial lavage with culture and the 32 species PCR-analyses
- Non-intubated patients Sputum or NPH culture with 32 species PCR-analyses
- Urine culture

*At deterioration during the ICU stay:*

- Blood culture with time to positivity from the CVC (all lumen) and the AC
- Intubated patients: Bronchial lavage with culture, 32 species PCR-analyses, Aspergillus-Ag (=galactomannan)
- Non- intubated patients: sputum with the same analyses as in the bronchial lavage.
- Urine cultures
- Possible wound cultures
- Fungal antigen (beta-glucan)
- Extended viral (i.e HSV, CMV, EBV) examinations can be performed in certain cases after discussion with the responsible infectionist.

Radiology and other examinations

- CT-thorax with analyses of Covid-19 specific findings and pulmonary embolus should be performed when transferring the patient to the ICU.
- A qualified (by a licenced operator) UCG should be performed within the first 24 hours. Assess myocardial function (left and right), valvular disease and pulmonary artery pressures.

**TRANSFER OF THE PATIENT TO THE ICU**

The postoperative unit is used as the Covid-ICU. The responsible physician should immediately call the head nurse for a brief report (phone 29131 or 22965) when decision for ICU admission is made.

The following data should be reported:

Patient data

- Short medical history
- Initial treatment strategy (including which medication and equipment that should be prepared)
- Initiation of treatment, placement of CVC and AC, and intubation is performed in COVID-19 ICU resuscitation area.

**TREATMENT:**

***Prescriptions:***

To facilitate the work in the ICU, all prescriptions should, as far as possible, follow standard drug regimen, standard doses and standard time (At 10 a.m. – 10 p.m., 10 a.m. -6 p.m. – 02 a.m. etc.). This is very time saving in the daily work. On demand prescriptions have shown to be difficult. If this method is used it has to be specified when and why it should be given.

***Respiration:***

Respiratory failure is the predominant symptom in severe Covid-19 disease. The patient should be transferred timely to the ICU if oxygen demand rapidly increases since deterioration to very severe illness can occur rapidly. The maximum degree of HFNO treatment at the wards is at FIO_2_ of 60% with a flow of 60 l/min. For logistic reasons, it is often convenient to perform the CT-scan after decision for ICU-admittance.

There are three flowsheets (Figure 1, 2 and 3) at the end of this document that give a basic structure for ventilatory strategies in Covid-19 patients on mechanical ventilation.

*High Flow Nasal Oxygen (HFNO) and Non-invasive ventilation (NIV)*

Many patients seem to respond well to HFNO. Read the departments separate documents for HFNO and NIV.

Humidification is probably of great importance. At the moment, it is unclear which patients benefit from either HFNO or NIV. We use HFNO as the primary treatment strategy. COPD patients might be an exception.

A patient without improvement or deterioration during the first two hours should be intubated. It is very important to recognize that HFNO and NIV can disguise deterioration. Especially young patients seem to be able to disguise clinical deterioration for a long time.

It is important to notice that the mentioned treatments give arise to viral aerosols. It is therefore mandatory to use full PPE. The aerosol production during HFNO treatment is comparable with oxygen treatment using a reservoir mask with flow of 15 l/min. It is now possible to give HFNO treatment at the Department of Infectious diseases with a flow of 40-60 l/min and a maximum FIO_2_ of 60%. If this treatment is insufficient, one should consider transfer to the ICU as soon as possible. This should also be considered for patient with high differences in FIO_2_ between prone and supine position.

Prone positioning during HFNO treatment will often improve oxygenation but it is not clearly shown that this strategy decreases the need for intubation or mortality. It has to be used with caution. Several days of HFNO in prone position can be very stressful for the patient and delay an inevitable intubation. It is also possible that prolonged treatment with a patient in respiratory distress can cause patient induced lung injury.

*Intubation:*

Several factors influence the decision on when to intubate: Consider intubation in these cases i:

- Rapid deterioration
- Insufficient effect effect of HFNO or NIV (SaO_2_ <88% despite treatment)
- Confusion
- Decreased level of consciousness
- Severe respiratory distress with high respiratory rate and intercostal retractions.

These recommendations are given from several centres treating Covid-19 ICU patients.

Intubation should be performed accordingly to the guidelines of the department with full PPE and a check-list. Consider using the Glidescope. If necessary during induction use a bag-valve mask ventilation with a HME approved for viruses.

The following medication should be prepared before induction:

- Inj Ketamine 10mg/ml 20 ml
- Inj Rocuronium 10 mg/ml 2 x 5 ml
- MS Propofol 20 mg/ml
- MS Fentanyl 0.05mg/ml
- MS Noradrenalin 40 microg/ml
- Inj Adrenalin 0.01 mg/ml 10 ml

Succinylcholine should be avoided due to prolonged inflammation.

*Mechanical ventilation:*

The majority of patients have, initially, normal or acceptable compliance. These patients have in the international literature been classified as L-patients (L: Low elastace = high compliance).

Lung injury seems to a large degree depend on micro thrombus formation within the lung (not classical pulmonary embolism from the legs) and decreased hypoxic vasoconstriction.

A minority of the patients will have a low compliance at arrival like the classical ARDS. These areclassified as H-patients (H: High elastance= low compliance).

A rough cut-off value of compliance between L- and H- patients is around 40 ml/cmH_2_0.

Ventilator treatment is often prolonged, at least one-two weeks. Our median time is 12 days.

- Use well-known respiratory modes- PCV, VCV, PRVC, and later on PS or VS. Avoid NAVA-ventilation since it is unclear how the lungs react to this mode and several of our new staff is unfamiliar with this treatment strategy.
- Most reports and international guidelines propose a modern ARDS-concept, which includes:

- - Tidal-volumes are prescribed from PBW (Predicted Body Weight). It is of high importance to set gender, length and age in the ventilator settings as soon as intubation has been performed. IT IS OF GREAT importance to avoid further lung injury.
  - L-patients:
    - PEEP ≤12 cmH_2_0 (Higher PEEP will probably give over distension and decrease preload even if oxygenation improves. Use as low PEEP as possible.
    - TV 7-8 ml/kg. If the driving pressure >15 H_2_0 decrease TV to 6 ml/kg.
    - Use balanced recruitment manoeuvres with peak pressures < 30 (40) cmH_2_0. Some patients will need higher peek pressures, especially after intubation.
  - H-patients
    - Individualize PEEP. Aim for PEEP <15 cmH_2_0, but higher PEEP might be necessary.
    - TV 5-6 ml/kg.
    - Use balanced recruitment manoeuvres with peak pressures < 40 cmH_2_0. Some patients will need higher peek pressures, especially after intubation
  - Increase Inspiratory/Expiratory quote (I/E). Measure auto-PEEP over time and static compliance during controlled ventilation.
- Try to get a driving pressure (Plateau pressure-PEEP) <15 cmH_2_O and peak pressure <30 cmH_2_O.
- Many patients seem to improve with mechanical ventilation in prone position. It should strongly be considered if FIO2 > 60% (or P/F ratio <15). Mechanical ventilation in prone position should be performed in controlled modes. The patient should be turned to supine position once a day for inspection of the skin, wounds, eyes etc. If deterioration is rapid in supine position than the prone strategy has to be initiated immediately. In other cases supine ventilation can proceed for several hours. Prone ventilation seems to decrease mortality even if oxygenation does not improve.
- Many patients are in the need for muscle relaxants for several days (sometimes weeks).
- Do not rush for supportive ventilationmodes! It takes time for the lungs to heal. In most cases there are also severe hyper-irritation and oedema of the mucus membranes in the respiratory tract.
  - Initiate supportive ventilation when FIO_2_ reaches 30% and PEEPr <10-12 cmH_2_O. Not sooner.
  - Decrease PEEP slowly
  - Aim for TV <10 ml/kg (PBW) during PS. Larger might increase the oedema formation and start new lung injuries.
  - To avoid new lung injuries, we have to start controlled ventilation again if the patient has severe stress during PS ventilation.
  - Aim for a P_0,1_ of 1,5 -5

There is no high-level evidence for routine use of inhalation treatment. It seems as iloprost and phosphodiesterase inhibitors (milrinone) could have some effect on pulmonary hypertension.

The effect of acetylcysteine seems to be very limited. Broncho dilatation therapy should only be used when there is clinical signs of bronchial obstruction.

ECMO should be considered in exceptional cases. Call the ECMO unit at Karolinska in Stockholm for discussion.

*Recruitment:*

Lung recruitment is often successful in the beginning of mechanical ventilation. Older recruitment technique with high PEEP and P_peak_ up to 60 cmH_2_0 or the “PEEP-method” should be avoided.

Be careful with recruitment manoevers in a ~~when~~ ~~the~~ patient with hypovolemia or ~~has~~ severe circulatory chock.

Recruitment manoeuvres should be performed after bronchoscopy, tracheal suctioning or other disconnections of the respiratory circle. Patients not responding to recruitment manoeuvres should not be recruited!

Recruitment manoeuvre suggestion # 1:

- Set PEEP at 10-15 cmH_2_0
- Increase P_peak_ stepwise to 30-40 cmH_2_0 (Until dynamic compliance decreases) during 40 seconds
- Repeat one or several times
- Follow dynamic compliance to find an optimal PEEP level to reduce the risk for atelectasis and overdistension

Recruitment manoeuvre suggestion # 2:

- Hold an inspiratory pause for 10 seconds
- Repeat

Recruitment manoeuvre suggestion # 3:

- Increase PEEP with 5 cmH_2_O for 5 minutes

*Extubation/reintubation:*

- Be cautious regarding premature extubation in Covid-19 patients. Be patient and do not extubate when CRP or PEEP are elevated.
- The patient must be fully awake, be free from anxiety, and have a calm respiration.
- Do always perform a cough-leakage test. The mucus membranes are often swollen which increases the risk for re-intubation. There is no data supporting any positive effect on inhalation with steroids or adrenalin.
- Reinubation is common. Reintubation should be performed as described above. Do not use succinylcholine

*Tracheostomy:*

There is no clear evidence for the optimal timing of performing tracheostomy. Decision has to be made on an individual basis. In our opinion, trachesostomy should be performed when the patient is clearly improving and is ~~which~~ is rarely the case before the need for prone positioning is over.. We believe that a tracheostomy facilitates weaning. Therefore, most of our patients will get a tracheostomy. Furthermore, transferal of cannulated patients to the ward will facilitate early care outside the ICU preparing space for patients in need for intensive care. An inner cannula should always be used when a patient is transferred to the ward with a tracheostomy and the patient always be reported to the ENT- Department.

*Humidification*

Almost all patients with severe Covid-19 infection will develop a lot of viscous mucous that will complicate mechanical ventilation. We have found that active humidification (and repeated bronchoscopies) are necessary to overcome this problem.

*Suctioning:*

1. Suctioning should only be performed when complicating ventilation or disturbing the patient`s ~~breathing efforts~~ ventilation

2. Perform a recruitment manoeuvre after each suctioning period (i.e. increasing the PEEP 5 cmH_2_O for around 5 minutes)

3. If it is large amounts of secretions or coagulated blood:

- Set the ventilator on stand-by
- Disconnect the tracheal tube and perform an adequate suction manoeuvre with a large-bore suction catheter (green or orange). Keep the hand in front of the tube opening as passive expiration can spread virus in the room.
- Connect the tube, start the ventilator and perform a recruitment manoeuvre.

*Bronchoscopy*

- Bronchoscopy should be performed hygienic as possible. Never reuse a single-use bronchoscope:
  - Always clean your hands and put on new gloves prior to bronchoscopy.
  - The bronchoscope should always be put on a sterile drape if there is a temporary pause in the procedure. Never put the bronchoscope on the blankets.
  - Use single-use containers for anti-fog liquid and silicone. One per patient.
- Bronchoscopy should always be performed in connection with intubation to secure microbiological samples (Bronchial lavage with culture and 32 species PCR-analyses)
- A new bronchoscopy should always be performed in suspicion of a new infection during mechanical ventilation. Perform lavage and order culture, 32 species PCR-analyses , and Aspergillus-Ag.
- The mucus production will almost always be severe after around 4-5 days on mechanical ventilation. It is therefore necessary with repeated bronchoscopies to clear the airways. This problem will often last for up to a week. The classical finding is a proximal white, very viscous mucus but a more clear mucus which is gluey attached to the mucus membrane in the distal bronchial tree.
- Every bronchoscopy has a downside with atelectasis and decreased surfactant. If performed recently a repeated bronchoscopy is unlikely to benefit the patient.
- The large-bore bronchoscopes (orange) are helpful in the proximal bronchial tree but cannot be advanced into the periphery as the green ones can.
- Always perform a recruitment manoeuvre after bronchoscopy.

***Cirkulation and fluids:***

- Hypovolemia (2-3l) is typical for most of the patientson arrival to the ICU. The patients are in need of optimal preload (especially the L-patients), and it is therefore important to prevent a negative fluid balance during the first days.Negative fluid balance will probably increase the risk for renal injury . Most of the patients will therefore have a limited positive fluid balance during the first days. Diuretics are rarely needed during the first days.
- Most of the patients present with cardiovascular stability but will be in need of noradrenalin to compensate for sedation.
- Almost all Covid-19 ICU patient present with an abnormal ECG:
  - Analysis of ECG on these patients is difficult. Especially for the cause of ECG changes.
  - Sinus tachycardia and a new atrial fibrillation are the most common types. The latter is often associated with myocarditis.
  - Malignant arrhythmias is almost always associated with myocarditis
  - Bradycardia and different AV-block are predominately seen in older patients. Most cardiac arrests are preceded with an AV-block
  - An elevated ST segment or a negative T-wave are common in many Covid-19 ICU patients without a simultaneous STEMI. It is probably difficult to differ between STEMI, myocarditis or general myocardial affection in these patients based on ST segment-evaluation.
- Strive after NORMOVOLEMIA with Plasmalyte® and Albumin. Do not try acheive normovolemia with high doses of noradrenalin.
- Several patients increase oxygenation levels after low doses of Dobutamine (2-5 microg/kg*min), independently of objective signs of heart failure.
- Right heart failure is common. Perform repeated UCGs. Treat with Dobutamine, phosphodiesterase inhibitors or levosimendan.
- In cases of pulmonary hypertension: try inhalation with phosphodiesterase inhibitors (milrinone) or iloprost.
- There are several descriptions on severe myocarditis and cardiogenic chock. We have seen some. The cause behind this in unclear and probably multifactorial. There are no curative treatments and there are few proposed treatment strategies but we have treated them ”as usual”. This means: Dobutamine, phosfodiesterase inhibitors and levosimedan, optimal heart rate, and normovelmia.
- Perform repeated UCGs and use Swan-Ganz catheter in severe cases.
- Several patient have brady- and tachycardia. We have induced several bradycardias (and asystole) with clonidine or dexmedetomidine. We have used isoprenaline with good effect in many patients. The need for a temporary pacemaker is increased in Covid patients as comparedto other ICU-patients.

Hypertension is very common in these patients. Especially during weaning. It can be difficult to separate from anxiety. Several patients have both problems. Treat hypertension as follows:

- Start ordinary chronic antihypertensive medication, especially beta-blockers. ACE-inhibitors and AII-2 blockers should be used with caution if the patient has a renal impairment or a high degree of inflammation.
- Acute lowering of BP: Inj Hydralazine 5 mg (sometimes infusion). Inj Trandate 20 mg iv could be used but are not as effective in Covid-19 patients..
- Longacting BP lowering:
  - T Carvedilol 12,5-25 mg x 2
  - T Metoprolol 25-50 mg x 3-4
  - T Amlodipine 5 mg x 1-2
  - T. Bisoprolol 10-20 mg

***Kidneys***

- Most of the patients will have micro thromb formation in the kidneys. It is also possible the the virus has a direct effect on the kidney cells. The kidneys are very sensitive to hypovolemia and the majority of the patients are dehydrated on arrival to the ICU. Be liberal with fluids and avoid diuretics during the first days (week). Strive for normovolemia!
- Rhabdomyolysis (see Rhabdomyolysis)
- A careful negative fluid balance can be achieved after the first days with single doses of furosemide or metolazone.

***Coagulation***

These patients are often in a hyper coagulative state which can be objectified with a ROTEM®-analysis. Increased D-dimer is typical. There are several reports on high incidences of DVT (perhaps up to 2/3) and pulmonary embolism. There are also extensive problems with micro thromb formation in different organs. Pulmonary and renal symptomps are predominant.

Pulmonary embolism should be suspected if there is a deterioration with hypoxia, right heart failure, gradually increasing D-dimer. Perform a CT-scan.

We use higher doses of tinzaparin as prophylaxis due to the hyper coagulation.

*Pharmacological prophylaxis and treatment:*

1. Dosage without CVVHDF: Body-weight <75 kg: tinzaparin 3.500 E x 2 sc, Body-weigt ≥75-90 kg: tinzaparin 4.500 E x 2 sc and >90 kg tinzaparin 75 E/kg x 2 sc.
2. Dosage with CVVHDF: tinzaparin 9.000- 12.000 E (375-500 E/h) via the syringe in the CVVHDF equipment
3. In cases of filter problems could tinzaparin be given sc and ileomedin be given iv.
4. In suspicion of pulmonary embolus: perform a CT-scan. If this not is possible, give tinzaparin in full dose, divided in two doses per day.
5. Heparin infusion: Consider three factors
   - Perform control of antifactor-Xa och anti-trombin
   - APT-time can be falsely low due to hyper inflammation.
   - APT-time can be falsely high due to hyper inflammation

Mechanical DVT prophylaxis should be considered in patients with contraindications to tinzaparin

*Monitoring of thrombo-prophylaxis:*

Monitor the effect of tinzaparin with antifactor-Xa. The response to a standard dose is very individual and several patients seem not to be adequately treated.

1. Anti-trombin: Consider substitution if anti-thrombin < 0.5kIE/l and try to reach >1.0 kIE/l
2. Antifactor-Xa (trough-level) the test should be performed right before a new dose and at least 3-5 doses have to be administered to reach steady-state (after first or a changed dose). The minimal time to first test during infusion therapy is 48 hours. Have a liberal approach to discuss anticoagulation matters with coagulation experts in Malmö.

Therapeutic target levels for antifactor-Xa:

- Prophylaxis trough level: 0.2-0.3 kIE/l
- Treatment trough level: 0.4-0,6 kIE/l

The trough level test should be performed within 30 minutes before a new dose is given.

*Anticoagulation and tracheostomy:*

The morning dose of tinzaparin should not be given on the day for surgical tracheostomy but administrated around two hours after surgery if no bleeding has occurred. If on continuous anticoagulation therapy no no interruption should be done but the ENT surgeon has to be informed.

***Stressulcusprofylaxis***

- Inj esomeprazole 40 mg iv x 1
- With established enteral nutrition: T Omeprazole 20 mg via gastric tube.

***Sedation and pain-management:***

We have experienced that patients with Covid-19 have been difficult to sedate during mechanical ventilation. If this is due to the illness and/or the situation is unclear. We have experienced that several of these patient need higher doses to accomplish adequate depth of sedation.

Sedation should be performed according to the departments’ routines and the depth of sedation should be prescribed every morning. Several patients will also need continous muscle relaxants for several days (weeks) during mechanical ventilation to accept the ventilator and prone position.

There are several reports on development of tolerance for all medications used for sedation in the Covid-ICU.

Respect the maximum dose for propofol (4 mg/kg*h) to avoid the *Propofol Infusion Syndrome* which is life-threating metabolic acidosis.

High blood pressure and tachycardia is not automatically an effect of under-sedation. It is often hypertension. The risk for awareness is very small with all medications given and many patients need antihypertensive drugs.

Clonidine and dexmetomedine should be used with caution in cases of bradycardia. We have seen several cases of asystole were these drugs have contributed.

- Sedation:
  - Step 1: give propofol after intubation
  - Step 2: After stabilisation and start of enteral nutrition: T lorazepam 1-2 mg x 3 and/or Mixt hydroxizine 50 mg x 2 to reduce the need for propofol (alternatively midazolam infusion)
  - Step 3: clonidine and dexmetomedine (not in cases of bradycardia or AV-block)
- Pain-management:
  - Step 1: fentanyl infusion
  - Step 2: mixt or iv oxycodone
  - Step 3: ketamine infusion

Avoid alfentanil due to the risk of bradycardia.

- Muscle relaxants: If the patient has difficulties in accepting the ventilator despite adequate sedation rocuronium should be administered. If possible-monitor TOF. TOF will probably help us to give a more optimal dose of rocuronium. Rocuronium should be withdrawn as soon as possible. If TOF measurements are not possible to perform it is of great importance to receive adequate relaxation. The patient should not interfere with ventilation at all.
- Paracetamol: Should only be given in cases of fever > 39℃ to reduce oxygen-consumption.
- Awakening: Give gradually decreased doses of sedation and define how it should be performed. On demand medication has turned out to be dysfunctional in this setting.

***Nutrition:***

- Nutrition assessment and delivery should be practiced accordingly to the department’s ordinary routines.
- After intubation: infusion of Glucose 10% Na/K.
- Avoid enteral nutrition in prone position. A low dose (10 ml/h) could be given if the patient is under hemodynamic control. Do not increase the dose until the patient is permanently in supine position. Medications can be given through the gastric tube in prone position.
- Enteral nutrition can be increased when the patient has established a supine position according to the routine of the department.
- If full enteral nutrition not is achieved after one week start supplemental parenteral nutrition
- Keep B-glucose < 11 mmol/l, preferably <8 mmol/l with continuous infusion of insulin. It is common with high doses of insulin due to steroid treatment and that many patient have diabetes mellitus type 2.
- Long lasting insulin could replace insulin infusion .at the end of the ICU stay.
- The amount of energy is difficult to decide. The energy demand is probably very low when the patient is deeply sedated and on muscle relaxants (perhaps 15-20 kcal per kg and day). Over-nutrition seems to be very dangerous in this situation. As the patient improves the energy- demand is probably higher (25-30 kcal per kg and day). It is difficult to know if measurements with the Quark® will give us more reliable knowledge. We have also seen that several patients have high urea but simultaneous low creatinine. We have not found out if this is a result of over-nutrition, protein catabolism, kidney failure or some other factor.

***Liver, pancreas and the GI-tract.***

- High LD is almost mandatory in patients with severe Covid-19 disease and a gradually increased level indicates severe illness. ASAT/ALAT and bilirubin is also often increased. However, liver failure seems to be rare.
- Micro thrombus in colon and colitis can occur. Use CT scan or colonoscopy for diagnosis. Discussions with a surgeon should be performed.
- Intestinal motility disturbances is very common. Obstipation is often the initial problem but it will evolve to diarrhoea after some days. Use motility-stimulating drugs and consider e double-lumen tube for gastric drainage and jejunal feeding in cases of prolonged gastroparesis.
- Several patients have paralytic obstruction of the small bowel. Exclude other pathology with a CT-scan. Use motility-stimulating drugs and occasionally neostigmine.
- Use a rectal tube with balloon in severe cases of diarrhoea.
- Increased amylase is very common but is most often subclinical. However, severe cases of pancreatitis is reported (even in Jönköping).

***Rhabdomyolysis:***

A limited rhabdomyolysis is common (P-myoglobin < 2.000-3.000 micrg/l). It is possible that this can increase the degree of kidney failure. The kidney failure should be prophylactic treated with normovolemia and crystalloid fluids. Maintain adequate diuresis (without furosemide)

A few patients will develop severe rhabdomyolysis. The mechanism is unclear but the reason could be micro thrombus formation in the muscles. P-myoglobin should be checked regularly, especially with decreasing urinary output and dark coloured urine. The treatment should be performed according to the routines of the department with forced diuresis and alkalinisation of the urine. CVVHDF seems not to have any protected effect but should be initiated (with high filtration levels) when the decreased urinary output cause clinical problems.

***Neurological problems:***

Covid-19 could affect the central and peripheral nervous system. The well-known first symptom is loss of taste and smell. There are also several reports on more life-threating situations. These are:

- Confusion
- Decreased level of consciousness
- Encelopathy
- Central micro thrombus formation
- Arterial and venous thrombus (especially in the posterior circulation of the brain). This includes even young persons.
- Intracerebral bleeding
- Epilepsy
- Guillain-Barre’s syndrome
- Demyelinisation (both central and peripheral)
- Encephalitis with covid-19. Reactivation of virus from the herpes group.

Virus can be found in the cerebrospinal fluid but there is no specific therapy.

It is growing evidence how Covid-19 can affect patient with chronical neurological diseases. Parkinsons disease seems not to be a risk factor by itself. About 40% of patients with Myasthenia Gravis (MG) are at risk for deterioration of their disease or a myasthenic crisis. Steroids can affect the MG symptomatology but should be given according to routines. Patients with MG require neurology consults..

***Anti-inflammatory treatment:***

Steroids:

- There is some evidence for corticosteroid use in covid-patients deteriorating and in need of oxygen therapy.
- Treatment with Betamethasone 6mg x 1 iv is initiated at the ward simultaneously with oxygen therapy
- Betamethason dosage in the Covid-ICU:
  - Bodyweight < 100 kg: Betamethasone 6 mg x 1 iv for 10 days (or longer)
  - Bodyweight 100-120 kg: Betamethasone 10 mg x 1 iv for 10 days (or longer)
  - Bodyweight ≥ 120 kg: Betamethasone 12 mg (or more) x 1 iv for 10 days (or longer)
  - Patients on chronic corticosteroid treatment need higher doses. All steroid administration should be intravenous.
- Clinical experience shows higher demands of corticosteroids (Betamethasone 12-18 mg iv) in some patients in order to reach sufficient effect. Decreasing CRP often notes 1-2 days after corticosteroid initiation.
- We have noticed that some patients react with rising CRP when suspending Betamethasone therapy. In thesecases we usually increase the dose again, especially if there is a clinical deterioration.
- It seems that some patients with severe ARDS are in need of corticosteroids for a longer time period. In these patients, length of steroid tapering is advised for a time period of several weeks, and occasionally months.
- Current evidence does not support the use of steroids in late or longstanding ARDS but may be considered in patients with low compliance (< 30ml/cmH_2_O) whitout improvement. In these cases start Methyprednisolone 125-250 mg x 1 iv. Taper over 10 days, sometimes longer.
- In summary, it is difficult to predict the patients` need for steroid use. Large inter-individual differences in need for steroids and treatment length seem to exist.

***Other anti-inflammatory treatments:***

- Tocilizumab is an IL-6 inhibitor. There is some evidence for early administration in quickly deteriorating patients and significant rise of inflammatory parameters (IL-6). Tocilizumab may potentially reduce the need for intensive care and shortens length of stay. Treatment with Tocilizumab is initiated pre-ICU by infectionists. Never in the ICU asit is too late.

Consider:

- Single dose treatment
- Patient needs to be on steroid treatment at least 48 hours prior to Tocilizumab administration.
- Contraindications: hematologic malignancy, immunosuppressive treatment, neutropenia, thrombocytopenia, recent intestinal perforation, diverticulitis, diverticulosis, gastric ulcer, pregnancy, hypersensitivity for Tocilizumab.
- Other reasons for deterioration have been excluded prior to dose: ie superinfection, pneumothorax, pulmonary embolus.
- CRP cannot be used for treatment guidance under 14 days after given dose.
- After given dose IL-6 needs to be checked day 3 and day 4. Discuss findings with infectionist.
- Steroid treatment needs to be continued 1-2 weeks after a Tocilizumab dose.
- Immunoglobulin and convalescent plasma therapy can be considered in individual cases. Discuss with infectionist.
- NSAID: NO!
- Statins: are not recommended for patients with Covid-19 but may be continued in patients normally using statin treatment if no rhabdomyolysis is present.

***Antimicrobial (bacterial and fungal) treatment:***

Be meticulous with bacterial cultures on patient arrival and on deterioration. This applies especially for bronchoalveolar lavage (BAL) or sputum.

Always take a BAL-culture, 32-agents PCR-test for lower airway including SARS-CoV-2, aspergillus-Ag from airway and beta-glucan if the patients deteriorates.

The majority of patients with severe covid-19 presents with high CRP (50-200mg/l) without any signs of bacterial secondary infections. Judgement concerning if the patient has a bacterial secondary infection based on CRP, pro-calcitonine and WBC is difficult (impossible). The inflammatory response differs significantly between patients regardless of secondary infection or not. Neutrophilic granulocytes are often increased and lymphocytes suppressed during Covid-19.

Judgement for initiation of empiric antibiotic therapy is difficult but the more severe Covid-19 infection the stronger the indication for antibiotic therapy. If antibiotic therapy is indicated,cefotaxime and erythromycine are recommended for initial treatment at intubation. Decision for continuation or discontinuation is made when results of BAL-cultures are available. We have noticed a high degree of simultaneous *H. influenzae* infection in the initial BAL-culture. Daily discussions with infectionist for possible adjustments and discontinuations of the antibiotic therapy are paramount.

Do not forget to discontinue antibiotic treatment when cultures are negative and to take new cultures if the patient deteriorates!

***Antiviral therapy:***

Remdesevir is approved by the Swedish Medical Products Agency for treatment of Covid-19. Limited access to remdesevir necessitates discussion with a infectionist prior to treatment commencement. It seems that remdesevir does not influence disease severity in patients already on mechanical ventilation. In conclusion, indications for remdesevir in Covid-19 ICU-patients are scarce.

Patients might be considered for remdesevir treatment if:

- Known immunodeficiency/immunosuppression. Especially with persistent viremia and oxygen demand.
- Patient without any signs of improvement.
- Persistent viremia

***Dermatology:***

Covid-19 seems to present with various skin lesions, i.e. erytema multiforme and erythema. We have noticed several patients with reactivation of herpes-infections in the skin and mucous membranes.

**Family members and relatives**

Due to the pandemic, ICU-visit-restrictions have been introduced for family members and relatives. This implies new ways of contact with family members and relatives:

- Information by telephone: the intensivist responsible for the patientshould inform one family member on a daily basis.
- Zoom, Skype, Facetime: seems to be a good alternative, especially when the patient is weaningfrom sedation. Aim for daily contact. This may be advisable even if the patient is in deep sedation.
- Visits of family members and relatives: visits have to be restricted and are indicated in three situations:

1. The patient is deteriorating and risk of death is high.
2. Extended length of stay in ICU.
3. With difficulties during awakening from sedation. We have noticed that the family members and relatives could have a very positive effect on psychological well-being during the weaning process. There is even some evidence supporting this practice.

**Contagiousness?**

Assessment of contagiousness is challenging in Covid-19 ICU-patients. Discussion with an infectionist and infection control physician is paramount and assessed all patients. Prior to that SARS-COV-2-antibody (IgG) analysis is mandatory. Decision about when patients are infection free is documented in the patient’s file by the infectionist.

<https://folkhalsaochsjukvard.rjl.se/dokument/evo/94985332-7fea-4faf-934d-360b8df21245?pageId=47070>

Figure 1: *Initial ventilation settings Covid-19 patient*

1. Start ventilator with PRVC, PC or VC
2. PEEP: 8-12 cmH_2_0
3. TV: 6-8 ml/kg PBW
4. RR: 18 per minute
5. Adjust FIO_2_  aiming for SaO_2_  92-96%
6. Adjust RR aiming for normoventilation
7. Aim for P_plat_ < 30 cmH_2_0
8. Aim for P_driv_ <15 cmH_2_0

After achieving hemodynamic stability:

1. Recruitment manoeuvrs up to 40 cmH_2_0.

Some patients might need higher pressure.

1. Assess static compliance and auto-PEEP

C_static_ <40ml ml/cmH_2_0

C_static_ >40ml ml/cmH_2_0

**H-patient**

Continue with figure 3

**L-patient**

Continue with figure 2

PRVC: Pressure Regulated Volume Control

PC: Pressure Control

VC: Volume Control

PEEP: Positive endexpiratory pressure

PBW: Predicted bodyweight

P_plat_: Plateau pressure

P_driv_: Driving pressure

C: Compliance

L: Low elastance

H: High elastance

Figur 2: *Ventilator settings Covid-19 patient with high compliance (L-type).*

1. Use PRVC, PC or VC
2. Adjust PEEP to reduce atelectasis, aim for PEEP < 12 cmH_2_0
3. TV 6-8 ml/kg PBW
4. RR adjusted according to pCO_2_ (normal)
5. Careful recruitment manoeuvres (P_top_ < 40 cmH_2_0)
6. Adjust FIO_2_ aiming for SaO_2_ 92-96%. Aim for higher I/E ratio
7. Measure auto-PEEP and C_stat_

If problems with high pressure:

P_plat_ > 30 cmH_2_0 eller

P_driv_ > 15cmH_2_0

If ventilation problems:

FIO_2_ rising to 60%, P/F ratio down to 15 and under

or pCO_2_ > 10-12 (pH<7,2)

1. Test inhalation of Milrinon or Iloprost
2. Check UCG. Right heart, left heart insufficiency?
3. Muscle relaxants
4. Prone position

Reduce TV to 5-6 ml/kg of PBW

Insufficient effect?

Insufficient effect?

PRVC: Pressure Regulated Volume Control

Contact with ECMO-unit for discussion!

PC: Pressure Control

VC: Volume Control

PEEP: Positive endexpiratory pressure

I/E: Inspiration/Expiration ratio

PBW: Predicted bodyweight

P_plat_: Plateau pressure

P_driv_: Driving pressure

P/F: ratio PaO_2_ and FiO_2_

C: Compliance

Stat: Static compliance

Figur 3: : *Ventilator settings Covid-19 patient with low compliance (H-type).*

1. Use PRVC, PC or VC

2. Adjust PEEP to reduce atelectasis, PEEP < 15 cmH20 (rarely higher)

3. TV 5-7 ml/kg PBW

4. RR adjusted by _P_CO_2_. Reduce dead-space at high pCO_2_

5. Careful recruitment manoeuvers (P_top_< 40 cmH_2_0). Omit if no previous effect

6. Adjust FIO_2_ aiming for SaO_2_ 92-96%. Aim for higher I/E ratio

7. Measure auto-PEEP and C_stat_

If problems with high pressure:

P_plat_ > 30 cmH_2_0 eller

P_driv_ > 15cmH_2_0

If ventilation problems:

FIO_2_ rising to 60%, P/F ratio down to 12

or pCO_2_ > 10-12 (pH<7,2)

1. Test inhalation with milrinone or Iloprost
2. Check UCG. Right heart, left/heart insufficiency?
3. Continous muscle relaxants
4. Prone position

Reduce TV to 5 ml/kg PBW

Insufficient effect?

Contact ECMO-unit for discussion!

PRVC: Pressure Regulated Volume Control

PC: Pressure Control

VC: Volume Control

PEEP: Positive endexpiratory pressure

I/E: Inspiration/Expiration ratio

PBW: Predicted bodyweight

P_plat_: Plateau pressure

P_driv_: Driving pressure

P/F: ratio PaO_2_ and FiO_2_

C: Compliance

Stat: Static compliance
