# Supplementary material for: Low mortality rates among critically ill adults with COVID‐19 at three non‐academic intensive care units in south Sweden
Source: Acta Anaesthesiol Scand. 2021 Sep 5;65(10):1457–65. doi: 10.1111/aas.13972 (PMC8441887; doi:10.1111/aas.13972)
Supplement: Supplementary file 2 — Supplementary Material [file AAS-65-1457-s002.pdf]

Region Jönköpings län

Behandlingsriktlinjer för patienter med

Covid-19

Op/IVA kliniken,

Länssjukhuset Ryhov.

ett patientnära förslag.

Version 3.4: 2021-05-03

Författare: Fredrik Hammarskjöld, Överläkare, Med Dr
Godkänd av: Thomas Öhman, Överläkare, MLA
Operations och Intensivvårdsavdelningen
Länssjukhuset Ryhov
Jönköping

BAKGRUND:

Patienterna med svår av Covid-19 infektion ställer oss in för nya utmaningar och till stor del saknas vetenskapligt stöd för att ge rekommendationer för behandling med hög evidence-grad. Dessa riktlinjer bygger på erfarenheter från andra länder men har efter hand kunnat anpassas efter våra egna erfarenheter och kunskaper i Sverige och Jönköping.

När det saknas bra forskning innebär det inte av vi kan pröva allt som vi tror är bra. Begreppet vetenskap och beprövad erfarenhet måste fortfarande vara det som styr vår behandling.

Eftersom inflödet av nya publikationer (sällan välgjorda studier utan redovisning av enstaka patienter eller begränsade fallserier) är stort kommer detta dokument att vara mycket dynamiskt och den aktuella versionen kommer hela tiden att finnas på klinikens hemsida. När en ny version presenteras kommer det skickas ut ett mail. Röd text markerar det som är nytt från förra versionen.

Det finns oftast många sätt att lösa ett problem. Resultaten för behandlingen av Covid-19 patienter på IVA i Jönköping har hittills varit mycket goda. En av de viktigaste anledningarna till detta är förmodligen en hög följsamhet till uppgjorda behandlingsrutiner. För att ytterligare höja patientsäkerheten och minska stressen hos våra medarbetare måste behandlingen göras så enkel och likriktad som det bara går. Detta gäller i synnerhet dessa patienter då alla medarbetare inte har intensivvårdsutbildning. Avancerade och tekniskt komplicerade behandlingar bör undvikas men kan vid enstaka tillfällen vara nödvändig.

Har ni synpunkter på dessa riktlinjer eller hittar nya behandlingsförslag kan ni gärna maila författaren.

VEM SKA TILL IVA:

Sedvanliga bedömningar för vem som förväntas ha nytta av intensivvård ska göras. Det sker en daglig kontakt med infektionskliniken eller annan enhet angående patienter som är aktuella för oss.

Om det är bestämt att intensivvård är aktuellt bör man vid tidig försämring av andning, dvs ökat syrgasbehov ex $SaO_2 < 90\%$ (trots $O_2 \geq 8$ liter på mask) eller $pCO_2 > 6$ kPa eller en stigande andningsfrekvens upp mot 30 per min, ta patienter med konstaterad Covid-19 till Covid-IVA då försämringen kan gå fort. Det är lätt att underskatta patientens andningsbesvär då de subjektivt mår bra trots högt syrgasbehov.

Övriga faktorer som ytterligare ökar indikationen för intensivvård är förvirring, medvetandepåverkan, dålig urinproduktion trots vätska, arytmier (exempelvis nydebuterat förmaksflimmer) eller cirkulatorisk instabilitet samt grundsjukdomar som ger sämre marginal att klara sig utan intensivvård. Låt inte patienterna bli för uttröttade på avdelningen och vi har sett några fall där patienten haft en väldigt ansträngd andning och att detta troligtvis har orsakat pneumothorax och pneumomediastinum.

Det börjar komma alltmer kunskap om riskfaktorer för svår infektion och mortalitet. Exempel på dessa är: hög ålder, låg fysisk funktionsgrad, hjärt-sjukdom, hypertoni, högt BMI, diabetes typ 2, KOL, rökning, dåligt immunförsvar, organtransplantation, behandling med immunmodulerande terapr (Ex Mabtera), manligt kön och eventuellt svår astma. Det har hittills varit svårt att visa att lindrigare lungsjukdomar (ex astma) är riskfaktorer.

Varje enskild patient måste dock bedömas enligt sina riskfaktorer vad gäller samsjuklighet, biologisk ålder, funktionsgrad och egen vilja till intensivvård. Eventuella behandlingsbegränsningar ska dokumenteras enligt sedvanliga rutiner (se separat arbetsbeskrivning).

FÖLJANDE LAB-ANALYSER INDIKERAR RISK FÖR SVÅR SJUKDOM OCH KANSKE ÄVEN MORTALITET.

- CRP >100 mg/l utan tecken på samtidig bakomliggande bakteriell infektion
- Successivt stigande LD till > 8 mikrokat/l
- Stigande kreatinin, gräns >100 mikromol/l hos tidigare njurfrisk individ
- Förhöjd D-dimer >1 mg/l FEU
- Troponin T > 15 nanog/L
- Låga lymfocyter (men normala eller höga leukocyter)
- S-Ferritin > 1000 microg/l

PROVTAGNING OCH UNDERSÖKNINGAR:

Kem. lab

Speciella Covid-19 menyer finns inlagd i BOS för Kem Lab, både vi ankomst och under vårdtid.

Mikrobiologiskt lab

Vid ankomst tas:

- Blododling
- Hos intuberad patient tas : Bronchodling, 32 luftvägsblock,
- Hos icke-intuberad patient tas: Sputum eller NPH odling samt 32-luftvägsblock från samma lokal
- Urinodling

Under vårdtid vid försämring eller oklar infektion eller inflammation:

- Blododling med tidsskillnad från både CVK (samtliga lumen) och AK
- Hos intuberad patient tas i BAL: Bronchodling, 32- block, Aspergillus-Ag (=galaktomannan)
- Hos icke-intuberad patient tas sputum (ev hjälp av sjukgymnast efter koksalt inahalation), med samma analys som i BAL
- NPH odling
- Urinodling
- Ev sårodlingar
- Svampantigen (beta-glucan)
- Eventuell vidare virusdiagnostik efter överenskommelse med infektionsläkare

Röntgen

- CT-thorax göras med frågeställning om infiltrat och lungemboli i samband med överflytt till Covid-IVA om detta inte är gjort innan. Om patientens tillstånd inte tillåter detta får det göras så fort det är möjligt efter intubation

Klin fys

- Inom första vårddygnet bör ett kompetent UKG göras med frågeställningarna: VK/HK funktion, vitium och tryck i lilla kretsloppet.

ÖVERFLYTT AV PATIENT TILL COVID-IVA

UVA används som lokal för intensivvård av Covid-19 patienter (se separat dokument). När beslut tas om att patienten ska till denna plats ska beslutande läkare ringa Teamansvarig sjuksköterska (T. 29131 eller 22965) och rapportera patienten.

Följande rapporteras enligt SBAR:

- Patientinformation
- Kort bakgrund
- Initiala åtgärder (inklusive MTU och läkemedel som ska förberedas)
- Vid ankomst till Covid-IVA läggs patienten UVA:s resusciteringsplats alternativt på IVA plats 10 eller 3 och flyttas sedan till lämplig plats.

BEHANDLING:

Läkemedelsordinationer:

För att underlätta arbetet inne på Covid-IVA bör man i möjligaste mån ordinera standardläkemedel i standarddoser på standardtider (ex kl 10-22, 10-18-02 osv). Detta är mycket tidssparande i det dagliga arbetet. Vid behovs medicinering har visat sig svårt. Om detta ska användas måste vad som är behov tydlig preciseras.

Andning:

Lungsvikt är oftast det dominerande symptomet vid svår COVID-19 sjukdom. Vid ökat syrgasbehov bör patienten tidigt tas till IVA för behandling då andningssvikten kan progrediera fort. Även om det är ett logistiskt problem bör CT thorax göras i tidigt skede och vid oförklarlig eller allvarlig försämring. Av logistiska skäl är det ofta bra att göra i CT i samband med att patienten överflyttas till Covid-IVA

I slutet av dokumentet finns tre flödesscheman som ger en grund för ventilatorstrategier hos Covid-19 patienter.

Högflödesgrimma och Non-invasiv ventialtion (NIV)

Många patienter verkar ha nytta av högflödesgrimma och eventuellt NIV. Läs respektive arbetsbeskrivning!

Förmodligen är befuktningen viktig jämfört med vanlig syrgas. Det är oklart vilka patienter som har bäst nytta av den högflödesgrimma eller NIV. I nuläget använder vi i första hand högflödesgrimma, med undantag för enstaka KOL-patienter.

Om patienten inte förbättras inom två timmer eller försämras bör man inte vänta med intubation förutsatt att det inte föreligger vårdbegränsningar. Det föreligger en risk att dessa behandlingar kan maskera försämring och att patienterna plötsligt blir väldigt snabbt försämrade. Framför allt unga patienter verkar kunna klara av ganska uttalad hypoxi under en längre tid och därigenom maskera en avsevärd försämring.

Observera att dessa behandlingar förmodligen ger aerosolbildning med virus och full skyddsklädsel måste alltid användas. Högflödesgrimma ger motsvarande mängd aerosol som syrgasbehandling med traumamask med ett flöde av 15 l/min. Patienter kan nu erhålla högflödesgrimma på infektionskliniken. Om det visar sig att denna behandling är otillräcklig och patienten behöver komma till Covid-IVA bör man vara restriktiv till fortsatt behandling med högflödesgrimma utan istället intubera patienten. För att behandling med högflödesgrimma ska vara effektiv bör flöden på 40-60 l/min användas. Om syrgasbehovet behöver överstiga 60% bör patienten, om den är aktuell för intensivvård, tas över till Covid-IVA. Detsamma gäller patienter där skillnaden mellan rygg-och bukläge är väldigt stor.

Buklägesventilation med högflödesgrimma förbättrar ofta syresättningen, men det är inte säkert att det minskar behovet av intubation eller minskar mortaliteten. Detta måste användas med omdöme. Flera dygns behandling på detta sätt är oerhört påfrestande för patienten och för lång väntan på intubation ska undvikas. Det finns också en risk att långdragen HFNO behandling kan ge lungskador som hade kunnat undvikas med tidig intubation.

Intubation:

Flera faktorer påverkar när intubation ska göras. Överväg intubation hos patienter med:

- Snabb försämring
- Dålig effekt eller utdragen behandling med högflödesgrimma eller NIV (SaO_2 <88% under längre tid trots intensiv behandling)
- Förvirring
- Sjunkande medvetandegrad
- Påtagligt ansträngd andning med hög andningsfrekvens och indragningar.

Detta förefaller vara en rekommendation som kommer från de flesta håll med erfarenhet av Covid-19 patienter. Använd i möjligaste mån en IVA-ventilator i samband med intubation.

Genomför detta enligt sedvanliga principer med full skyddsutrustning. I möjligaste mån genomförs detta av två läkare. Använd i först hand glidescope för att öka avståndet från ditt eget ansikte till patientens luftväg. Försök genomföra RSI i apné men är detta inte möjligt ur hypoxi-avseende får naturligtvis maskventilation genomföras. Maskventilation ska ske med virussäker HME (passiv befuktare).

Inför varje intubation förbereds följande läkemedel:

- Inj Ketalar 10mg/ml 20 ml
- Ing Rocuronium 10 mg/ml 2 x 5 ml
- MS Propofol 20 mg/ml
- MS Fentanyl 0.05mg/ml
- MS Noradrenalin 40 microg/ml
- Inj Adrenalin 0.01 mg/ml 10 ml

Celocurin bör undvikas då patienterna ofta har varit inflammatoriska under en längre tid.

Respiratorbehandling:

Observera att majoriteten av dessa patienter initialt har en normal eller bra compliance och i de flesta fall ej ska behandlas som vanliga ARDS-patienter, dessa har i internationell litteratur kommit att kallas L-patienter (L: Low elastace = hög compliance).

Lungskadan förefaller bero på mikrotromber i lungan som ofta ej är embolier från DVT:er i benen utan bildas lokalt i små lungkärl samt en hämmad hypoxisk vasokonstriktion).

Enstaka patienter kommer från början att ha lågt compliance eller utveckla detta pga sjukdom eller behandling till en mer klassisk ARDS-bild och kallas H-patienter (H: High elastance= låg compliance).

Skillnaden mellan L- och H- patienter dras vid en compliance på ca 40 ml/cmH_20.

Respiratorbehandlingen är ofta långdragen. Räkna med 1-2 (minst!) veckor i respirator.

- Använd välkända respiratorsätt, dvs TK, VKTS, VK och senare TU och VU. Undvik NAVA-ventilation då det är oklart hur dessa lungor reagerar på detta men framför allt att många av de som nu behandlar patienterna helt saknar vana vid denna behandlingsmodalitet.
- De flesta rapporter och internationella riktlinjer förespråkar modernt ARDS koncept vilket innebär:

- Tidalvolymer ordineras utifrån beräknad idealvikt (PBW= Predicted Body Weight). Det är väldigt viktigt att ni snarast lägger in patienten kön, uppmätta längd och ålder i respirator/narkosapparat för att detta ska bli rätt. OBS! Superviktigt för att inte skada lungan!
- L-patienter:
  - PEEP ≤12 cmH_20 (Högre PEEP kommer sträcka sönder lungan även om syresättning blir bättre och försämra preload). Sträva efter så låga värden som möjligt.
  - TV 7-8 ml/kg. Om drivtryck >15 H_20 sänk TV till 6 ml/kg.
  - Måttlig rekrytering med topptryck < 30 (40) cmH_20. Enstaka patienter kan kräva högre tryck, speciellt efter intubation.
- H-patienter
  - Individualiserat PEEP. Sträva efter värden <15 cmH_20 men det kan krävas högre. Individualisera.
  - TV 5-6 ml/kg.
  - Måttlig rekrytering med topptryck <40 cmH_20. Enstaka patienter kan kräva högre tryck, speciellt efter intubation.
- Förlängd Inspirations/Expirationskvot (I/E) kvot. Följ auto-PEEP och statisk compliance vid kontrollerad andning.

- Patienterna svarar ofta bra på rekrytering tidigt i förloppet.
- Försök att ha drivtryck (platåtryck-PEEP) <15 cmH_2O. och topptryck <30 cmH_2O.
- Många patienter svarar bra på buklägesventilation. Om FIO_2 stiger upp mot 60% (eller P/F kvot <15) bör detta starkt övervägas. Buklägesventilation bör ske i kontrollerat mode. Patienten bör vändas tillbaka på rygg en gång per dygn för kontroll av hud, trycksår etc. Blir patienten hastigt försämrad får man påbörja buklägesventilation tämligen omgående igen. I annat fall är det bra om ventilation på rygg/sida kan ske under några timmar. Buklägesventilationen verkar minska mortaliteten oavsett om patienten får en förbättrad syresättning eller ej under behandlingen.
- Många patienter kräver intermittent eller kontinuerlig muskelrelaxation under flera dagar och uppväckningsbekymmer är vanliga.
- Skynda långsamt. Det tar lång tid för lungan att läka. Ofta kvarstår dessutom en påtaglig irritation och svullnad av luftvägsslemhinnorna.
  - Övergå inte till understödd andning förrän FIO_2 närmar sig 30% och PEEP är <10-12 cmH_2O.
  - Sänk PEEP långsamt.
  - Vid TU bör man sikta på TV <10 ml/kg (utifrån PBW). Större TV ökar risken för ny ödembildning i lungan och nya lungskador.
  - Om patienten har en stor ansträngning för att klara spontanandning måste man ta ett steg tillbaka till kontrollerad ventilation. I annat fall ökar risken för nya lungskador.

Det finns i nuläget inga rapporter om att inhalationsbehandling rutinmässigt ska användas. Det förefaller som om Ventavis och fosfodieterashämmare kan ha viss effekt. Acetylcystein har begränsad effekt på Covid-19 patienternas sega sekret och bronchdilaterare kan ordineras på enskilda patienter efter kliniskt behov.

Enstaka patienter kan bli aktuella för ECMO behandling.

Rekrytering

Lungrekrytering förefaller ofta framgångsrikt i början av sjukdomen. Tidigare rekrytering till topptryck upp mot 60 cmH_20 och rekrytering med PEEP-metod avrådes ifrån.

Var försiktig med rekrytering om patienten är hypovolem eller svårt hemodynamiskt instabil.

Efter bronchoscopi, sugning eller annan isärkoppling av andningscirkel är patienten ofta i behov av rekrytering. Patienter som ej svarar på rekrytering ska inte utsättas för denna manöver

Rekryteringsförslag 1:

- Sätt PEEP på 10-15 cmH_20
- Höj luftvägstryck så att Ptopp blir 30-40 cmH_20 i 40 sekunder
- Återupprepa en eller flera gånger
- Följ dynamisk compliance för att finna bästa PEEP och minska risken för översträckning av lungan.

Rekryteringsförslag 2:

- Håll förlängd inspiratorisk paus i ca 10 sekunder
- Upprepa efter behov

Rekryteringsförslag 3 (efter sugning):

- Höja PEEP med 5 cmH_2O under ca 5 min

Extubation/reintubation:

- Det är väldigt lätt att extubera för tidigt. Ha tålamod och extubera aldrig vid högt CRP eller höga PEEP. Patienten bör vara ordentligt vaken, ångestfri och ha en lugn andning för att extubation ska var framgångsrik.
- Genomför alltid cough-leakage test. Slemhinnorna är ofta svullna vilket påtagligt ökar risken för reintubation. Det finns inga data som stöder att detta undviks med steroider eller inhalation med adrenalin.
- Reinubation är tyvärr vanligt. Detta görs enligt våra intubationsrutiner. Undvik celocurin.

Tracheostomi:

Optimal tidpunkt för tracheostomi är oklart utan får bedömas från fall till fall, men bör inte göras förrän patienten är i klar förbättring och *helst när buklägesventilation ej längre behövs*. Under Corona-pandemin har vi kommit att tracheostomera majoriteten av respiratorpatienterna då vi upplever att detta högst påtagligt underlättar urträning. Tracheostomier genomförs inne på Covid-IVA med öppen teknik av öronläkare. Ett flertal av patienterna överförs till vårdavdelning efter IVA med tracheostomi pga av slemproblem och larynxsvullnad. Använd då innerkanyl och rapportera patienten till öronkliniken.

Om patientens tillstånd tillåter bör patienten vara i apné (med respirator i viloläge) under själva incision av trachalringarna samt nedförande av trachealkanyl. I övrigt görs detta enligt gällande arbetsbeskrivning och med full skyddsutrustning av alla.

Befuktning

Majoriteten av patienterna utvecklar en omfattande slemproblematik med ett vitt mycket segt sekret. Aktiv befuktning (och upprepade bronchoscopier) är ofta nödvändigt.

Sugning:

Tänk på:

1. Sugning ska endast göras när det påverkar ventilationen eller stör patienten. Ej pga av biljud.

2. Rekrytera efter sugning (exempelvis med att höja PEEP 5 cmH_2O under ca 5 min)

3. Vid mycket slem eller koagler kan man tvingas suga gör så här:

- Stäng av respiratorn (=vänteläge)
- Lossa vid tuben och sug ordentligt med grov sugkateter (grön eller orange). Håll handen för så att inte den passiva expiration skapar aerosol ut i rummet.
- Koppla ihop respiratorcirkeln igen och starta ventilationen. Rekrytera (exempelvis med att höja PEEP 5 cmH_2O under ca 5 min)

Broncoscopi

- Broncoscopi ska göras så rent det bara är möjlig och alltid ett nytt engångsbroncoscop för varje undersökningstillfälle:
    - Sprita händerna och ta på nya rena handskar
    - Broncoscopet läggs på steril engångsduk innan och under en ev paus i undersökningen.
    - Använd engångsbehållare med anti-fog och silikon (en för varje patient)
- Broncoscopi ska alltid göras efter intubation för att säkerställa mikrobiologisk diagnostik. Ta (BAL med standardodling, 32-luftvägsblock)
- Vid misstanke om ny infektion bör BAL med standardodling, 32-luftvägsblock och Aspergillus-Ag göras.
- Upprepade broncoscopier är ofta nödvändiga efter ett antal dagar i respirator när slemproduktionen kommer igång. Proximalt ses ett väldigt segt sekret som är vitt och mer distalt ett klart mindre segt sekret. Gör hellre en ordentlig rengöring än flera per dygn.
- Varje broncoscopi har en baksida med minskad surfactant och atelektaser. Är patienten nyligen broncoscoperad och försämras igen är det osannolikt att en ny scopi hjälper.
- Det grövsta engångsbroncoscopen (orange) är bra att suga proximalt i luftvägarna men kommer inte ut distalt till det perifera klara sekretet vilket det gröna gör.
- Rekrytering ska alltid göras efter broncoscopi

Cirkulation och vätska:

- Majoriteten av patienterna har förlorat 2-3 liter vätska när det kommer till IVA. I synnerhet L-patienterna är väldigt beroende av preload varför man oftast inte ska sträva efter negativ vätskebalans under de första dygnen. Det riskerar även att skada njurarna. Räkna med att patienten behöver gå i en plusbalans under de första dygnen. Under de första dygnen bör man vara mycket restriktiv med diuretika.
- De flesta av patienterna är förhållandevis cirkulatoriskt stabila men kräver noradrenalin för sedering.
- Nästan alla patienter med intensivvårdskrävande Covid har EKG förändringar:
    - Tolkning av EKG hos Covid-IVA patienter är svårt. Detta gäller framförallt genesen till förändringarna.
    - Sinustackycardi och nydebuterat förmaksflimmer är vanligast och det senare indikerar ofta ett myocarditinslag
    - Maligna arytmier är nästan alltid associerade med myocardit
    - Bradycardi och olika AV-block ses framför allt hos de äldre. De flesta hjärtstopp föregås av AV-block
    - Påverkad ST-sträcka och T-negativiseringar förekommer hos många Covid-IVA patienter. Det anses inte gå att särskilja STEMI, myocardit eller generell myocardpåverkan med hjälp av EKG.
- Sträva som alltid efter normovolemi med Plasmalyte och Albumin (återhållsamhet om Albumin är > 30g/l). Försök inte åstadkomma normovolemi med höga doser noradrenalin.
- Flera patienter förbättrar syresättningen med en lågdos Dobutamin (2-5 microg/kg*min) när de är som sjukast oavsett om UKG avslöjar vänster- eller högerkammarsvikt
- Högersvikt är vanligt. Gör upprepade UKG. Behandla med Dobutamin, Milrinon eller Simdax.
- Vid pulmonell hypertension kan man med fördel prova kontinuerlig inhalation av milrinon eller ventavis
- Det finns flera beskrivningar om svår myokardit och svår kardiogen chock. Enstaka fall har setts i Jönköping. Det är oklar genes till detta och någon specifik behandling finns ej. Det finns heller inte några regelrätta förslag på hur detta ska hanteras utan det verkar som om man får göra ”som vanligt”, dvs använda Dobutamin i första hand, och därefter Simdax och Corotrop samt optimera hjärtfrekvensen.
- Patienterna bör följas med UKG och pro-BNP.
- Flera av patienterna har haft både tachy- och bradycardier. Vi har sett flera fall där Dexdor och Catapresan ytterligare bromsat hjärtfrekvensen och tom givit upphov till asystoli som krävt HLR. Det förefaller som behovet av pacemaker i denna patientgrupp är högre jämfört med vanliga IVA-patienter. Inne på Covid-IVA har vi en egen transvenös pacemaker.

Det är mycket vanligt med hypertoni bland dessa patienter, speciellt i tillfriskningsfas. Det kan vara svårt att skilja detta mot själslig stress och ofta förekommer bägge problemen samtidigt. Hypertoni kan behandlas enligt följande:

- Återinsätt ordinarie hypertonimedicin, framför allt om det är betablockad. ACE-hämmare och angiotensin-2 blockerare kan också återinsättas, speciellt i tillfriskningsfas men var försiktig vid njurpåverkan och påtaglig inflammation.
- Akut sänkning av blodtrycket: Inj Nepresol 5 mg och eventuell infusion är ofta bra. Trandate 20 mg iv tills önskad effekt åstadkommits kan också användas men är ofta inte lika effektivt.
- För mer långsiktig blodtrycksänkning finns följande alternativ:
    - T Carvedilol 12,5-25 mg x 2
    - T Seloken 25-50 mg x 3-4
    - T Amlodipin 5 mg x 1-2
    - T. Bisoprolol 10-20 mg

Njurar

- De flesta patienter verkar få mikrotromber i njurarna och eventuellt finns det även en direkteffekt av virus på njurarna. De är väldigt känsliga för hypovolemi och majoriteten av patienterna är dehydrerade vid ankomst till IVA. Ge frikostigt vätska och undvik diuretika under de första dygnen (veckan). Patienten bör alltid vara normovolem.
- Rhabdomyolys (se nedan)
- Försiktig negativ vätskebalans ges efter hand med hjälp av enstaka doser furosemid.

Koagulation

Dessa patienter är oftast hyperkoagulerande vilket kan objektiviseras med en ROTEM analys. Förhöjd D-dimer är vanligt hos de med allvarlig sjukdom. Det kommer allt flera rapporter om hög DVT frekvens (kanske upp till 2/3) och lungembolisering (kanske upp till 1/4). Det finns dessutom en omfattande problematik med mikrotrombotisering i flera organ där symtom från lungor och njurar oftast är dominerande. Generellt ökad blödningsrisk är ovanlig.

Vid försämring, dvs sämre syresättning, oförklarlig hypoxi, nytillkommen högersvikt eller fortsatt ökande D-dimer ska lungembolisering tidigt misstänkas.

Pga av detta ska vi ge högre dos trombosprofylax.

Farmakologisk trombosprofylax och behandling

1. Dosering utan PRISMA-behandling: Kroppsvikt <75 kg: Innohep 3.500 E x 2 sc, Kroppsvikt ≥75-90 kg: Innohep 4.500 E x 2 sc och >90 kg ges Innohep 75 E/kg x 2 sc.
2. Dosering med PRISMA-behandling: Innohep 9.000- 12.000 E (375-500 E/h) via PRISMAS motorspruta
3. Om det är problem med dålig filteröverlevnad i PRISMA filter kan man komplettera subcutan dosering Innohep med Ileomedininfusion via PRISMAN.
4. Om misstanken om lungembolisering uppkommer ska det om möjligt göras CT-med kontrast. Är detta inte möjligt så bör patienten behandlas med fulldos Innohep, uppdelat på två doser per dygn.
5. Heparin infusion: det finns två saker att vara medveten om i Covidsammanhang:
   - Kontroller Antifaktor-Xa och anti-trombin
   - APT-tid kan ge falskt lågavärden vid hyperinflammatoriska tillstånd. Detta innebär att patienten kan ha fullgod effekt trotts att inte terapeutiskt intervall uppnåtts.
   - APT-tid kan ge falskt höga värden vid hyperinflammatoriska tillstånd. Detta kan innebära att patenten är otillräckligt antikoagulerad trots förlängd APT-tid

Hos patienter som inte kan få fulldos av innohepprofylax ska venpumpsstövlar användas.

Monitorering av trombosprofylax:

Var mycket liberal med att monitorera effekten av Innohep med antifaktor-Xa. Det är svårt att förutsäga effekten av en viss dos hos Covid-19 patienter och ett flertal patienter blir underbehandlade.

1. Anti-trombin: Överväg substitution vid bristande effekt om värdet är < 0.5kIE/l och försök nå en nivå över 1.0 kIE/l
2. Antifaktor-Xa: Dalvärde tas men man måste vänta 3-5 doser (från behandlingsstart eller ändrad dos) innan steady-state har uppnåtts innan prov tas. Vid infusion tas prov först efter 48 timmar av konstant behandling. Diskutera gärna svar med koagulationsexpertis.

Behandlingsmål för antifaktor-Xa:

- Trombosprofylax dalvärde: 0.2-0.3 kIE/l
- Behandlingsdos dalvärde: 0.4-0,6 kIE/l

Dalvärde tas inom 30 min för nästa dos eller när som helst om infusion varit konstant i > 48 timmar.

Antikoagulation och tracheostomi:

Inför tracheostomi bör man hoppa över morgondosen med Innohep och ge den ca två timmar efter att operationen är genomförd. Kvällsdosen ges sen som vanligt. Vid kontinuerlig Innohepinfusion bör uppehåll helst ej göras. Öronläkare ska dock informeras om detta.

Stressulcusprofylax

- Inj Nexium 40 mg iv x 1
- Vid fungerande GI-kanal kan T Omeprazol 20 mg ges via sond.

Sedering och smärtbehandling:

Det har visat sig att patienter med Covid-19 har varit svårsederade i respirator och kräver förhållandevis stora doser läkemedel för att uppnå önskat sederingsdjup.

Sedering sker enligt kliniken vanliga principer och sederingdjup ska ordineras vid varje rond. Tänk på att de efter intubation ofta behöver vara relaxerade och djupt sederade under en längre tid (flera dygn-veckor) för att acceptera respiratorn och eventuellt bukläge.

Det finns flera beskrivningar om toleransutveckling av de sederande läkemedlen (särskilt Dexdor) varför ett visst växelbruk bör ske hos en patient som vårdas under längre tid,.

Överdosera inte Propofol (maxdos 4 mg/kg*h). Det finns risk för *Propofol Infusion Syndrome* vilket innebär en livshotande och svårbehandlad metabol acidos.

Högt blodtryck och tachycardi innebär inte med automatik för lite sedering utan är ofta en ren hypertoniproblematik. Risken att dessa svårt sjuka patienter med flera olika sederings- och smärtstillande läkemedel har awereness är mycket liten. Många patienter behöver behandling mot högt blodtryck.

Var försiktig med Catapresan och Dexdor vid bradycarditendens. Dessa patienter är mycket känsliga för detta och vi har sett flera hjärtstillestånd pga av detta.

- Sedering:
  - Steg 1: När patienten läggs i respirator. Grundsedering med Propofol.
  - Steg 2: Efter stabilisering och fungerande GI-kanal: T Temesta 1-2 mg x 3 och/eller Mixt Atarax 50 mg x 2 för att minska Propofolbehovet.
  - Steg 3: Catapresan eller Dexdor (inte vid bradycardi eller AV-block!)

- Smärtbehandling:
  - 1:a hand Fentanylinfusion.
  - 2:a hand Mixt eller Injektion Oxynorm x 6, Ketalarinfusion

Undvik Ultiva pga av bradycardirisken

- Muskelrelaxantia: Om patienten är svårventilerad ges enstaka doser rocuronium eller infusion efter bolus. Använd ev TOF-mätare och ge minsta möjliga mängd rocuronium för att minska risken för critical-illness polyneuropati. Oftast behöver TOF < 2. Saknas TOF-mätare ska infusions ges så att patienten är tydligt slapp och ej interagerar med respiratorn. När patientens situation börjar stabiliseras bör man göra regelbundna försök att minska eller sätta ut muskelblockaden.

- Paracetamol: Ges endast om feber > 39°C för att minska syrekonsumtionen.

- Uppväckning: Ge successivt minskande doser och skriv tydliga uttrappningsschema. Vid behov medicinering har hittills inte fungerat så bra.

Nutrition:

- Detta bör ske enligt nuvarande rutiner för kliniken.
- Initialt får patienten Glukoslösning med Na/K.
- Undvik sondmat i bukläge. Om patienten är cirkulatoriskt stabil kan 10 ml/h timma ges men öka inte. Tabletter kan ges.
- När patienten har etablerat ryggläge, dvs. sannolikheten för nytt bukläge är liten, startas eller ökas sondmat enligt vanlig rutin
- De patienter som ej har full enteral nutrition efter 1 vecka ska ha komplettering med parenteral nutrition.
- B-glukos måste ligga under 11 mmol/l, helst under 8 mmol/l. Ofta krävs relativt höga doser insulin då många har typ II diabetes och samtidigt får kortison.
- Om patienten har insulin i motorspruta så överväg att sätta över patienten på Insulatard sc med intermittent tillägg av Insulin Lispro iv vb. Detta gäller i synnerhet när patienten närmar sig utskrivning från IVA.
- Mängden energi är svår att avgöra. Om patienten är djupt sederad och muskelrelaxerad är förmodligen energibehovet lågt (exempelvis 15-20 kcal per kg och dygn). I denna situation är det verkligt farliga övernutrition. Vid tillfriskning är behovet säkerligen högre (exempelvis 25-30 kcal per kg och dygn), men återigen svårvärderat. I nuläget är det osäkert om Quarkmätning tillför något. Vi ser många patienter med lågt kreatinin men högre urea. Om detta är ett uttryck för övernutrition, proteinkatabolism, njursvikt eller något annat är oklart.

Lever och tarm:

- Förhöjt LD verkar vara väldigt vanligt hos dessa patienter och stigande värden indikerar allvarlig sjukdom. Det är vanligt med förhöjda transaminaser samt bilirubin. Regelrätt leversvikt förefaller ovanligt.
- Mikrotrombotisering i colon med colit är väl beskrivet. Diagnos görs med CT/scopi och kontakt tas med kirurg.
- Tarmmotilitetsstörningar är vanliga. Initialt ses obstipation och sedan diarréer. Använd motilitetsstimulerande läkemedel men glöm inte att minska och ev sätta ut dessa efter hand. Överväg stay-put sond om det är problem med ventrikeltömningen
- Flera patienter har paralytisk tunntarmsileus. Uteslut annan tarmsjukdom med CT. Behandla med motilitetsstimulerare och eventuellt enstaka doser neostigmin.
- Använd avföringsslang vid diarré då flera patienter utsöndrar virus via faeces.
- Pankreatiter orsakade av Covid-19 förekommer (även i Jönköping). Hos många ses en ökning av amylas men hos enstaka utvecklas en klinisk pankreatit.

Rhabdomyolys:

Rhabdomyolys förekommer ofta av lindrig grad (P-myoglobin < 2.000-3.000 micrg/l) hos många patienter med Covid-19. Möjligtvis kan detta bidraga till njursvikt och bör förebyggas med normovolemi och god diures mha av kristalloida vätskor.

Enstaka patienter kan få svår rhabdomyolys med betydligt större muskelcellssönderfall. Möjligtvis beror även detta på mikroembolisering och det kan komma både tidigt och sent i sjukdomsförloppet. Pga av detta bör vi kontrollera P-myoglobin med regelbundenhet, speciellt vid sviktande diures och mörkfärgad urin.

Behandlingen är forcerad diures och ev alkalinisering av urinen (se separat arbetsbeskrivning). Prisma verkar inte ha något protektiv effekt men startas vid sviktande diures som ger kliniska problem. Förslagsvis då med CVVHDF med förhållandevis hög filtrationsandel.

Neurologiska problem :

Covid-19 kan påverka både det centrala- och perifera nervssystemet. Välkända första symptom är försämrad smak och lukt. Det finns flera fallbeskrivningar på mer allvarliga och livshotande symptom. Dessa är:

- Förvirring
- Medvetandesänkning
- Encephalopati
- Microtromber
- Arteriella och venösa tromboser (speciellt i bakre cirkulationen). Även hos unga personer.
- Intracerebral blödning
- Epilepsi.
- Guillain-Barrés syndrom
- Demyelisering (både centralt och perifert)
- Covid-19 kan orsaka encephalit. Reaktivering av herpesgruppens virus med efterföljande encephalit förekommer också.

Virus kan påvisas i liquor men det saknas specifik behandling utan terapin bör helt vara inriktad på understödjande åtgärder utifrån symptom.

Hur Covid-19 infektioner påverkar specifika neurologiska sjukdomar är inte helt säkert men det finns nu data som visar att Parkinsonpatienter klarar sjukdomen bra och ca 40% av patienter med Myastenia Gravis (MG) risker en försämring i sin grundsjukdom eller drabbas av myasten kris. Steroider kan påverka MG men ska ges enligt sedvanlig Covid-19 rutin. Om MG försämras tas kontakt med neurolog för att diskutera behandling med immunoglobulin.

Antiinflammatorisk behandling:

Steroider:

- Det finns i nuläget ett visst vetenskapligt stöd för steroidbehandling av Covid-patinter när de försämras så att de behöver syrgas.
- Infektionskliniken påbörjar behandling med Inj Betapred 6 mg x 1 iv.
- Vid ankomst till Covid-IVA ges:
  - Kroppsvikt < 100 kg: Inj Betapred 6 mg x 1 i 10 dagar
  - Kroppsvikt 100-120 kg: Inj Betapred 10 mg x 1 i 10 dagar
  - Kroppsvikt ≥ 120 kg In Betapred 12 mg (eller mer) x 1 i 10 dagar
  - Vid kronisk steroidbehandling måste dosen anpassas men i allmänhet måste högre doser ges och alltid intravenöst.
- I vår kliniska vardag upplever vi att vissa behöver en högre dos (t.ex Betapred 12-18 mg x 1 iv) för effekt. Ofta ses en CRP nedgång 1-2 dygn efter insättande av kortison.
- Vi har också sett att en del patienter stiger i CRP och blir försämrade när Betapred sätts ut.
- Vid svår lungsjukdom verkar det som behandlingstiden bör förlängas och en successiv urtrappning bör ske under flera veckor.
- I nuläget rekommenderas ej steroider på indikationen sen ARDS eller ARDS som är långvarig men kan övervägas i enstaka fall med låg compliance (< 30ml/cmH_2O). Då ges exempelvis Solu-Medrol 125-250 mg x 1 som trappas ut på ca 10 dagar. De kan ibland behöva en lägre uttrappande steroidbehanling under längre tid.
- Sammantaget är det svårt att säga mer än patienterna mår bra av tidiga steroider i måttliga doser men det verkar finnas en stor individuell variation på behov och behandlingslängd.

Övrig antinflammatorisk behandling

- Roactemra ® (Tocilizumab) är en IL-6 hämmare. Det finns ett visst vetenskapligt stöd att ge detta tidigt i Covidsjukdomen vid hastiga försämringar och rejält stigande inflammatoriska parametrar (speciellt IL-6). Preparatet kan ev förhindra intensivvård och ev förkorta vårdtiden. Tocilizumab har framför allt sin plats på avdelning innan IVA och ordineras alltid av infektionsläkare i grupp.

  Att tänka på:

  - Preparatet ges som engångsdos
  - Patienten måste haft steroidbehandling i minst 48 timmar innan behandlingen ges
  - Kontraindikationer: Hematologisk malignitet, Immunsuppressiv behandling, Neutropeni, Trombocytopeni, nyligen tarmperforation, Divertikulit/Divertikulos, Aktivt ulcus, Överkänslighet mot Tocilzumab och graviditet.
  - Annan orsak till försämring ska ha uteslutits, ex bakteriell superinfektion, pneumothorax och lungemboli.
  - Efter given dos kan ej CRP användas för analys under ca 14 dagar
  - Efter given behandlingsdos kontrolleras IL-6 Dag 3 och Dag 4
  - Steroidbehandling bör fortgå upp till 1-2 veckor efter given dos.

- Immunoglobuliner och konvalecentplasma kan ges i enstaka fall. Detta ska endast ges efter noggrann diskussion med infektionsläkare.
- NSAID: nej.

- Statiner: Rekommenderas ej som generell medicinering vid Covid-19 men patienter som normalt använder dessa preparat ska fortsätta under pågående Covid-19 infektion om inte rhabdomyolys föreligger.

Antimikrobiell (bakterier och svamp) behandling:

Var noga med att ta adekvata odlingar vid ankomst och försämring. Detta gäller i synnerhet BAL eller sputum. Gör **både** odling, 32-luftvägsblock (inkluderar Covid-prov), samt Aspergillus-diagnostik från luftvägar (Aspergillus-Ag) och beta-glucan vid försämring.

Majoriteten av patienterna med svår sjukdom har höga CRP (50-200 mg/l) utan att ha en bakteriell sekundärinfektion. Det är mycket svårt att utifrån CRP, Pro-kalcitonin och LPK avgöra huruvida patienten har en sekundär bakteriell infektion. Det inflammatoriska svaret varier stort oavsett sekundär infektion eller ej. Neutrofila granulocyter är ofta förhöjda och lymfocyter sänkta av Covid-sjukdomen. i sig.

Indikationen för empirisk behandling vid intubation är svår men ju allvarligare patientens sjukdom är desto starkare är indikationen. I nuläget startas Cefotaxim och eventuellt Erytromycin. Eventuell utsättning eller justering görs efter svar från BAL-diagnostik. Vi har sett att många av våra patienter har samtidig infektion med *H. Influenzae* vid ankomst till IVA. Insättande av antibiotika samt justering av detta ska alltid ske tillsammans med infektionsläkare. Glöm inte att sätta ut antibiotika om odlingar är negativa och ta nya odlingar vid försämring.

Antiviral terapi:

Remdesevir är nu godkänt av Läkemedelsverket för behandling av Covid-19. Tillgången på läkemedlet är begränsat och all användning *ska alltid* diskuteras med infektionsläkare. Det förefaller inte påverka förloppet hos de som redan hamnat i respirator och följaktligen är våra patienter oftast inte aktuella för Remdesevir.

De patienter som framför allt kan vara aktuella är:

- Känd avancerad immundefekt/immunsuppression, Speciellt vid pågående viremi och/eller syrgasbehov
- Patient som inte förbättras och risk för intensivvård är överhängande
- Patient med kvarstående viremi

Hud:

Covid-19 förefaller kunna ge en mängd olika hudsymptom, exempelvis olika erytematösa förändringar men även erytema multiforme. Vi har sett ett flertal patienter med reaktivering av herpes-infektioner på hud och slemhinnor

ANHÖRIGA

Pga av Covid-19:s smittsamhet och Covid-IVA lokalens utformning kan vi inte erbjuda anhörigbesök som vi brukar att gör på IVA. Detta gör att vi måste arbeta annorlunda:

- Telefoninformation: Den ansvarige läkaren ska varje dag ring och informera en anhörig om den aktuella situationen. Vid en allvarlig försämring ska även anhöriga informeras
- Zoom, Skype, Facetime: verkar vara ett bra alternativ, speciellt när patienterna är i uppvakningsfas. Sträva efter daglig kontakt om det är möjligt.
- Det kan även vara bra för anhöriga när patienten är som sjukast, trots att patienten är nedsövd.
- Besök av anhöriga: Besöka av anhöriga är tillåtet i begränsad omfattning och det gäller framför allt vid tre tillfällen:
    1. Patienten är så sjuk så att risken för död är akut överhängande
    2. Väldigt långdraget vårdförlopp
    3. Vid svår uppväckning. Vi har sett att många med delirium vid uppvaknandet får det lättare om anhöriga finns på plats. Det finns även en viss vetenskap som stöder detta nu.

NÄR ÄR MAN SMITTFRI?

Detta är inte fråga som har ett absolut svar och blir särskilt svår hos intensivvårdspatienter, i synnerhet om de är immunsupprimerade. För varje patient måste detta göras på individuell grund. Denna diskussion bör göras under kontorstid tillsammans med infektionsläkare som är väl bekanta med patienten (eventuellt även diskussion med Smittskyddskliniken). Inför denna diskussion ska Klin kem-provet: *SARS-COV-2-ak (IgG)(tidigare infektion)* vara taget.

Beslut om smittfrihet ska dokumenteras av ansvarig infektionsläkare i patientjournal.

På denna länk hos Smittskydd och Vårdhygien finns dokument som styr bedömningen av smittfrihet.

https://folkhalsaochsjukvard.rjl.se/dokument/evo/94985332-7fea-4faf-934d-360b8df21245?pageId=47070

Figur 1: *Initial respirator behandling av Covid-19 patient*

1. Starta ventilation med VKTS, VK eller TK
2. PEEP: 8-12 cmH$_2$0
3. TV: 6-8 ml/kg PBW
4. Andningsfrekvens: 18 per minut
5. Styr FIO$_2$ så att SaO$_2$ är 92-96%
6. Styr andningsfrekvens efter pCO$_2$
7. Sikta på $P_{platå}$ < 30 cmH$_2$0
8. Sikta på P_{driv} <15 cmH$_2$0

När patienten börjar bli hemodynamisk stabil

1. Utför rekrytering upp till max 40 cmH$_2$0. Enstaka patienter kan behöva högre.
2. Mät statisk compliance och auto-PEEP

$C_{statisk}$ >40ml ml/cmH$_2$0

Patienten är **L-patient**

Gå till figur 2

$C_{statisk}$ <40ml ml/cmH$_2$0

Patienten är **H-patient**

Gå till figur 3

VKTS: Volymkontrollerad tryckstyrning
VK: Volymkontroll
TK: Tryckkontroll
PEEP: Positive endexpiratory pressure
PBW: Predicted bodyweight
$P_{platå}$: Platåtryck
P_{driv}: Drivtryck
C: Compliance
L: Low elastance
H: High elastance

Figur 2: *Respiratorbehandling av patient med Covid-19 med god compliance (L-typ).*

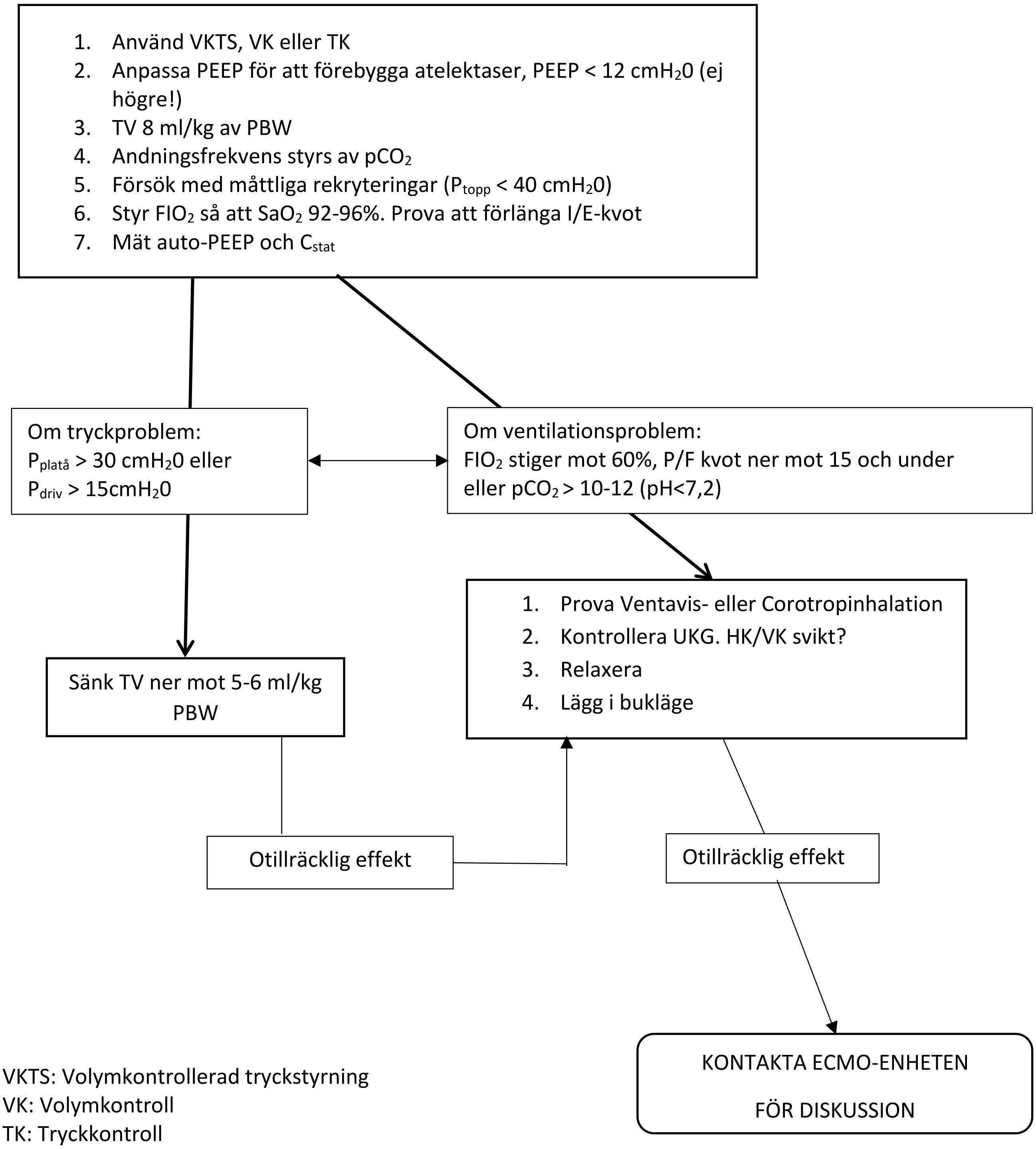

VKTS: Volymkontrollerad tryckstyrning
VK: Volymkontroll
TK: Tryckkontroll
PEEP: Positive endexpiratory pressure
I/E: Inspiration/Expiration
PBW: Predicted bodyweight
$P_{platå}$: Platåtryck
P_{driv}: Drivtryck
P/F: kvoten mellan PaO_2 och FiO_2
C: Compliance
Stat: Statisk
HK/VK: Höger-/vänsterkammare

Figur 3: *Respiratorbehandling av patient med Covid-19 med låg compliance (H-typ).*

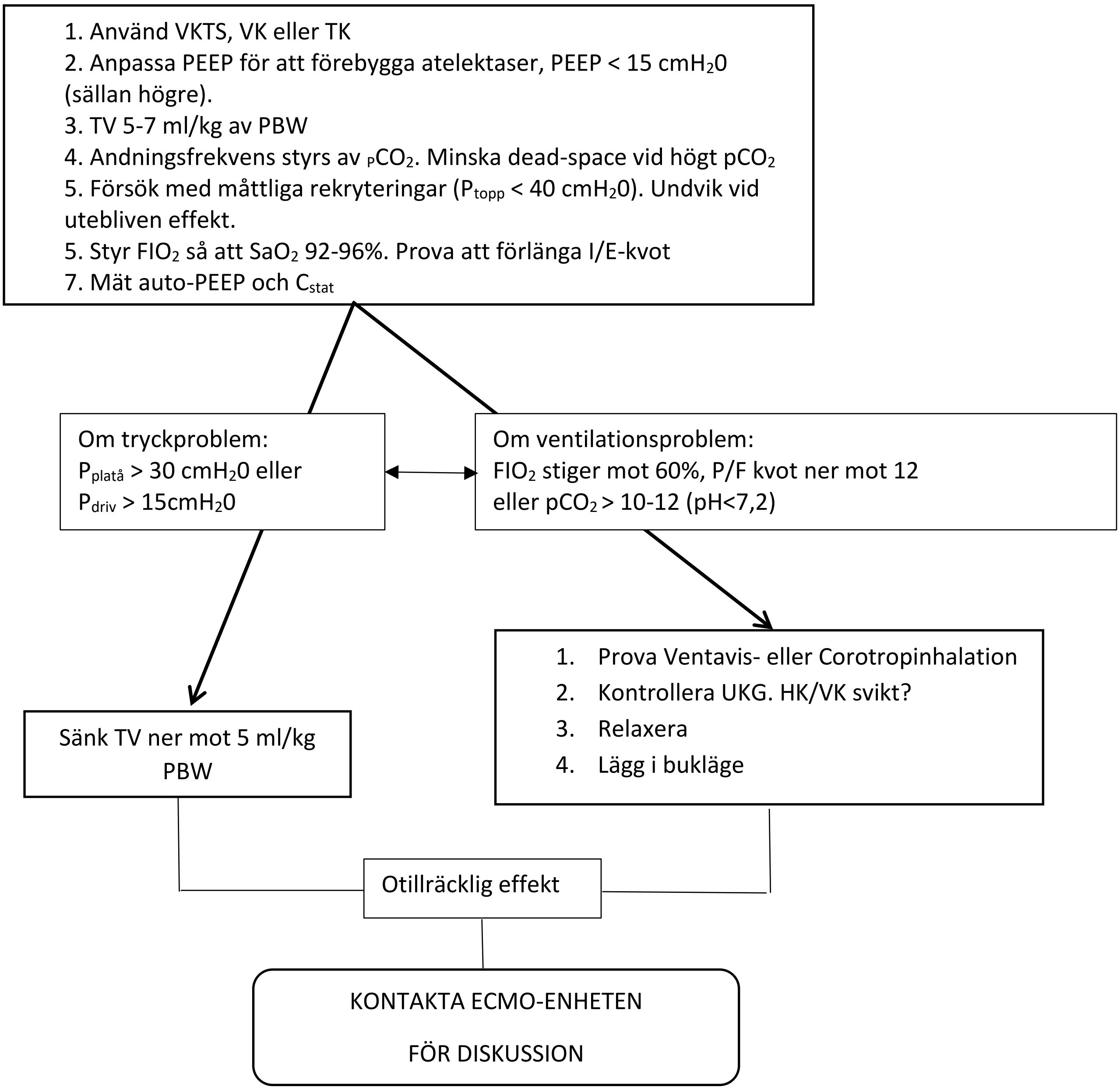

VKTS: Volymkontrollerad tryckstyrning
VK: Volymkontroll
TK: Tryckkontroll
PEEP: Positive endexpiratory pressure
I/E: Inspiration/Expiration
PBW: Predicted bodyweight
$P_{platå}$: Platåtryck
P_{driv}: Drivtryck
P/F: kvoten mellan PaO_2 och FiO_2
C: Compliance
Stat: Statisk
HK/VK: Höger-/vänsterkammare
